# Time-of-flight resolved light field fluctuations reveal deep human tissue physiology

**DOI:** 10.1038/s41467-019-14228-5

**Published:** 2020-01-20

**Authors:** Oybek Kholiqov, Wenjun Zhou, Tingwei Zhang, V. N. Du Le, Vivek J. Srinivasan

**Affiliations:** 1 Department of Biomedical Engineering, University of California Davis, Davis, CA 95616 USA; 2 Department of Ophthalmology and Vision Science, University of California Davis, Davis School of Medicine, Sacramento, CA 96817 USA

**Keywords:** Near-infrared spectroscopy, Optical physics, Optical spectroscopy

## Abstract

Red blood cells (RBCs) transport oxygen to tissues and remove carbon dioxide. Diffuse optical flowmetry (DOF) assesses deep tissue RBC dynamics by measuring coherent fluctuations of multiply scattered near-infrared light intensity. While classical DOF measurements empirically correlate with blood flow, they remain far-removed from light scattering physics and difficult to interpret in layered media. To advance DOF measurements closer to the physics, here we introduce an interferometric technique, surmounting challenges of bulk motion to apply it in awake humans. We reveal two measurement dimensions: optical phase, and time-of-flight (TOF), the latter with 22 picosecond resolution. With this multidimensional data, we directly confirm the unordered, or Brownian, nature of optically probed RBC dynamics typically assumed in classical DOF. We illustrate how incorrect absorption assumptions, anisotropic RBC scattering, and layered tissues may confound classical DOF. By comparison, our direct method enables accurate and comprehensive assessment of blood flow dynamics in humans.

## Abstract (web summary)

Diffuse optical flowmetry is widely used to assess blood flow dynamics, but has remained difficult to interpret. Here, the authors use interferometric near-infrared spectroscopy, revealing additional information about optical phase and time-of-flight, and observe the Brownian nature of blood flow dynamics in humans.

## Introduction

The separation and characterization of light paths in random media are a goal in many fields, including colloid science, fiber-optic communications, and biophotonics. Near-infrared spectroscopy (NIRS) endeavors to assess deep biological tissue in vivo by measurements of highly scattered near-infrared light, which can penetrate centimeters beneath the surface before detection. NIRS measures either incoherent or coherent light intensity. Incoherent NIRS includes continuous wave (CW-) NIRS^1^, the simplest technique, as well as more advanced (temporal) frequency domain^2,3^, spatial frequency domain^4^, and time-domain (TD-)^5,6^ NIRS approaches. Coherent NIRS aims to assess dynamics of turbid media through fluctuations of multiply scattered light^7^. In particular, diffusing wave spectroscopy/diffuse correlation spectroscopy (DWS/DCS) probes red blood cell (RBC) dynamics related to blood flow deep in biological tissues from coherent light intensity fluctuations^8^.

Measuring blood flow in tissues such as the brain^9,10^ is paramount, and methods of diffuse optical flowmetry (DOF) including DWS/DCS and related coherent techniques (e.g. laser Doppler^11^ and laser speckle^12^) are widely used^8^. In the diffuse scattering regime, relevant for probing deep tissue, while the theory connecting scatterer dynamics to light fluctuations is established^13^, these methods remain largely empirical, without a proven physical model for RBC motion (i.e. advection or Brownian motion). There are two major reasons for this disconnect. First, classical DWS/DCS cannot resolve light dynamics in time-of-flight (TOF), though efforts are underway to improve TOF discrimination^14–16^. Second, though DWS/DCS autocorrelation decay times correlate empirically with blood flow^17^; counterintuitively, the functional form of observed autocorrelations resembles theory for Brownian motion, not random flow^8^. At intermediate source–detector (SD) separations, these issues are further confounded by the fact that single scattering with Brownian motion and diffuse scattering with random flow both yield similar TOF-integrated intensity autocorrelations^18,19^. Numerical simulations of correlation transport theory in synthetic vascular beds support the Brownian motion model in larger vessels with laminar flow^20^. However, existing in vivo TOF-integrated measurements are simply too coarse to assess competing physical models, as many models fit experimental observations^21,22^.

**Fig. 1:**
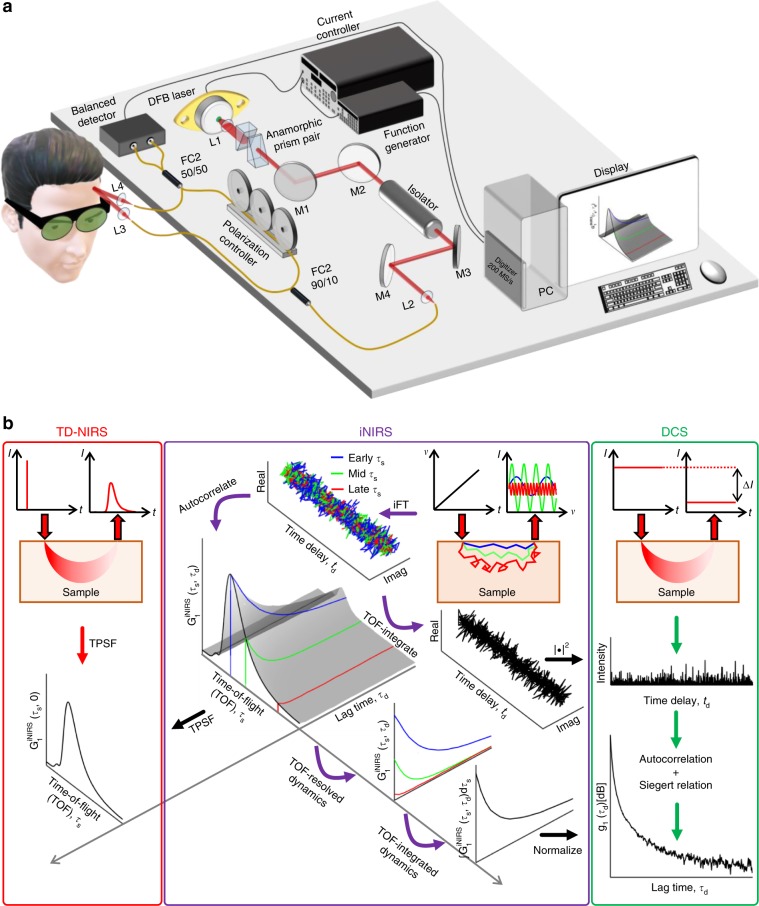
Interferometric near-infrared spectroscopy (iNIRS). **a** iNIRS recovers TOF-resolved light distributions and dynamics by rapidly wavelength tuning a temporally coherent distributed feedback (DFB) laser. **b** iNIRS (center) provides a rich two-dimensional data set, ${{G}}_1^{{{{\rm{iNIRS}}}}}$(τ_*s*_, τ_*d*_), that unifies incoherent and coherent diffuse optics. Through incoherent averaging, ${{G}}_1^{{\mathrm{iNIRS}}}$(τ_*s*_, 0) provides medium temporal point spread functions (TPSFs) like time domain (TD-) NIRS (left). Through time-of-flight (TOF) integration, ${\int} {{{G}}_1^{{\mathrm{iNIRS}}}}$(τ_*s*_, τ_*d*_)dτ_*s*_ can provide medium autocorrelations, like diffuse correlation spectroscopy (DCS) (right).

Here, we advance an optical method called interferometric near-infrared spectroscopy (iNIRS)^23^ for human use (Fig. 1a), thus bringing in vivo diffuse light scattering experiments closer to the underlying physics (Fig. 1b). In contrast to coherence-gated methods that attempt to isolate singly scattered, superficial light^24^, iNIRS embraces multiply scattered, deeply penetrating light, while assessing it quantitatively. By tuning the light source optical frequency, iNIRS efficiently^25^ and directly measures intrinsic field autocorrelations with 22–45 ps TOF resolution and shot noise limited sensitivity^26^. After carefully estimating and correcting extrinsic motion artifacts to isolate intrinsic tissue dynamics, in all studied tissues, we discern multiple exponentially decaying autocorrelation components, each with a distinct TOF distribution. By deconstructing DWS/DCS, we then illustrate how these TOF-resolved components contribute to classical TOF-integrated autocorrelations in DOF. We verify our interpretations through multi-modality experiments, with scattering contrast agents, in different species, and during physiological manipulations. Our detailed data sets enhance understanding of light scattering dynamics in biological tissues, enabling more accurate monitoring of blood flow based on sound physical underpinnings.

## Results

### In vivo bulk motion correction

Non-contact assessment of optical field fluctuations in humans requires management of motion artifacts. A uniform axial sample velocity imparts a linear phase shift to the sample field, and thus, the estimated field autocorrelation. Consequently, if the sample velocity changes randomly during the time window for autocorrelation estimation, sample dynamics are overestimated (see Supplementary Note 1).

**Fig. 2:**
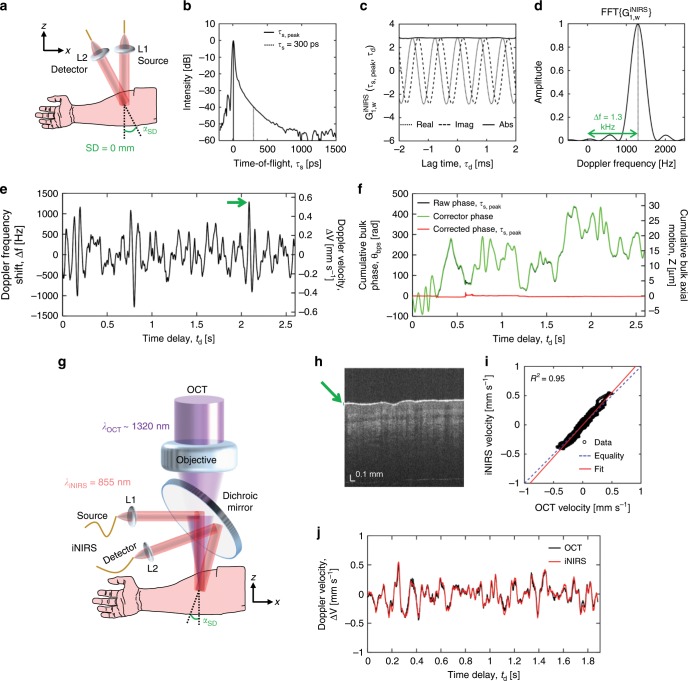
Correction of sample motion enables field-based diffuse optics. **a** Geometry for human forearm measurement, where α_SD_ (source–detector angle) is ~10°. For a 2 ms segment at the peak time-of-flight (solid black vertical line in **b**), the autocorrelation (**c**) rotates with lag time τ_*d*_, implying a linear phase shift due to sample motion. **d** The rate of phase change, i.e. the Doppler frequency shift, can be calculated from the power spectrum (Fourier transform of the autocorrelation ${{G}}_{1,{{w}}}^{{\mathrm{iNIRS}}}$) of the segment. **e** The Doppler frequency shift time course can also be expressed as a Doppler velocity ∆V assuming α_SD_ = 0°. **f** Motion correction using the estimated phase (green) virtually eliminates phase drift at the peak TOF, as seen by comparing black and red curves. As shown in Supplementary Fig. 2, phase correction also restores the validity of the modified Siegert relationship. **g**–**j** Simultaneous comparison of bulk phase shifts due to axial motion in iNIRS and optical coherence tomography (OCT). **g** The two systems are combined with a dichroic beam splitter such that ~1320 nm OCT light is transmitted, while 855 nm iNIRS light is reflected. **h** OCT cross-sectional image from the combined system; Doppler velocity to estimate bulk motion is calculated from the first 50 µm from the skin surface as shown by the green arrow. The Doppler velocity parametric plot (**i**) and time courses (**j**) show excellent agreement between modalities (*R*^2^ = 0.95).

To solve this, we employ short SD separations to provide a large backscattered/few-scattered static reference from which phase drift can be estimated and subsequently corrected. To maintain sufficient dynamic range to detect long TOF diffuse light at short SD separations, we apply digital spectral shaping (Supplementary Fig. 1). To assess motion, we consider the iNIRS field autocorrelation, ${{G}}_{1,{{w}}}^{{\mathrm{iNIRS}}}$, at null SD separation in the human forearm, estimated over a short 2 ms window, at τ_*s*,peak_, the TOF of the temporal point spread function (TPSF) maximum (Fig. 2a, b). The behavior of ${{G}}_{1,{{w}}}^{{\mathrm{iNIRS}}}$, where the real and imaginary parts are sinusoidal and in quadrature (Fig. 2c), corresponds to rotation in the complex plane, which implies a Doppler frequency shift, ∆*f* = ∆θ_bps_/(2π∆τ_*d*_), where ∆θ_bps_ and ∆τ_*d*_ are bulk Doppler phase shift and lag time resolution, respectively. Therefore, we assume a uniform Doppler velocity over this short window. The Doppler phase/frequency shift can be approximated from the peak of the Fourier transform of ${{G}}_{1,{{w}}}^{{\mathrm{iNIRS}}}$ (power spectrum, Fig. 2d). The Doppler frequency shift time course over a 2.5 s acquisition is then obtained by sliding the short window across the time course (Fig. 2e). We assume that the peak TOF for null SD consists largely of backscattered light from extravascular tissue. This assumption is reasonable as backscattered light in a single mode is typically orders of magnitude larger than multiply scattered light in that mode^27^, and the fractional blood volume of most tissues is typically on the order of 5% or less^28,29^. As the sole intrinsic dynamics of static tissue are slow intracellular motility, we propose that this Doppler shift is caused by bulk axial motion of the sample. The bulk Doppler phase shift is summed cumulatively over time and unwrapped to yield the cumulative unwrapped phase, θ_bps_. Conversion from cumulative unwrapped phase to axial shift *Z* based on *Z*(*t*_*d*_) = θ_bps_(*t*_*d*_)λ_0_/(4π) suggests micrometer-scale bulk motion (solid black line in Fig. 2f, right axis). As bulk motion should be identical for all TOFs, it can also be determined by integrating ${{G}}_{1,{{w}}}^{{\mathrm{iNIRS}}}$ over TOF prior to phase estimation (green in Fig. 2f). Upon correction (Supplementary Fig. 2), the cumulative bulk phase θ_bps_ at the peak TOF is nearly constant, as expected for static tissue (red in Fig. 2f).

Furthermore, rotation in the mutual coherence function is eliminated by motion correction (Supplementary Fig. 2a). As the intrinsic field autocorrelation is expected to be real for unbiased dynamics, we take the real part of the autocorrelation after phase correction as our estimate of ${{G}}_1^{{\mathrm{iNIRS}}}$. We also verify that the imaginary part consists of noise. The correction methodology is further validated by examining ${{G}}_1^{{\mathrm{iNIRS}}}$ at selected TOFs. Prior to correction, the field autocorrelation at τ_*s*,peak_ (solid black in Supplementary Fig. 2b) decays in ~700 μs, more than an order of magnitude faster than the ${{G}}_2^{{\mathrm{iNIRS}}}$ decay time^25^, whereas the heterodyne Siegert relationship^25^ predicts that ${{G}}_2^{{\mathrm{iNIRS}}}$ should decay faster than ${{G}}_1^{{\mathrm{iNIRS}}}$. This apparent discrepancy occurs because the estimated field autocorrelation is corrupted by bulk phase shifts (see Supplementary Note 1). The autocorrelation of the bulk phase shift estimate (green in Supplementary Fig. 2b) reveals that indeed, the unexpectedly fast decay is explained by motion. Phase correction increases the decay time at τ_*s*,peak_ to ~200 ms (solid red in Supplementary Fig. 2b), while its impact at τ_*s*_ = 300 ps (~35% increase) is less striking due to the higher intrinsic decay time, but still significant. As shown in Supplementary Fig. 2c, the intensity autocorrelation predicted from the iNIRS field autocorrelation approaches the directly-determined intensity autocorrelation, showing that phase correction restores the validity of the modified Siegert relationship (see Supplementary Note 2)^25^.

### Co-registered validation against OCT

Validation of our phase drift estimation was performed via simultaneous co-registered comparison (Fig. 2g) with a gold standard technique, OCT, where bulk phase shifts due to motion are routinely estimated from static tissue and corrected^30^. A standard OCT cross-section of forearm tissue reveals epidermal and dermal layers (Fig. 2h), consisting mainly of intrinsically static tissue from which OCT bulk phase shifts can be estimated^30^. In particular, the calculated OCT Doppler velocity over the axial range in the epidermis, indicated by a green arrow, with no vasculature, should yield bulk motion. A parametric plot of Doppler velocities over 1.9 s, ∆*V* = ∆*f*λ_0_/2 (Eq. (4) with α_SD_ = 0°), and accounting for different center wavelengths, yields excellent correlation *R*^2^ = 0.95 (Fig. 2i) and agreement (Fig. 2j), conclusively showing that iNIRS bulk phase drift results from sample axial motion.

### Phase correction significantly impacts field autocorrelation decays at early TOFs

**Fig. 3:**
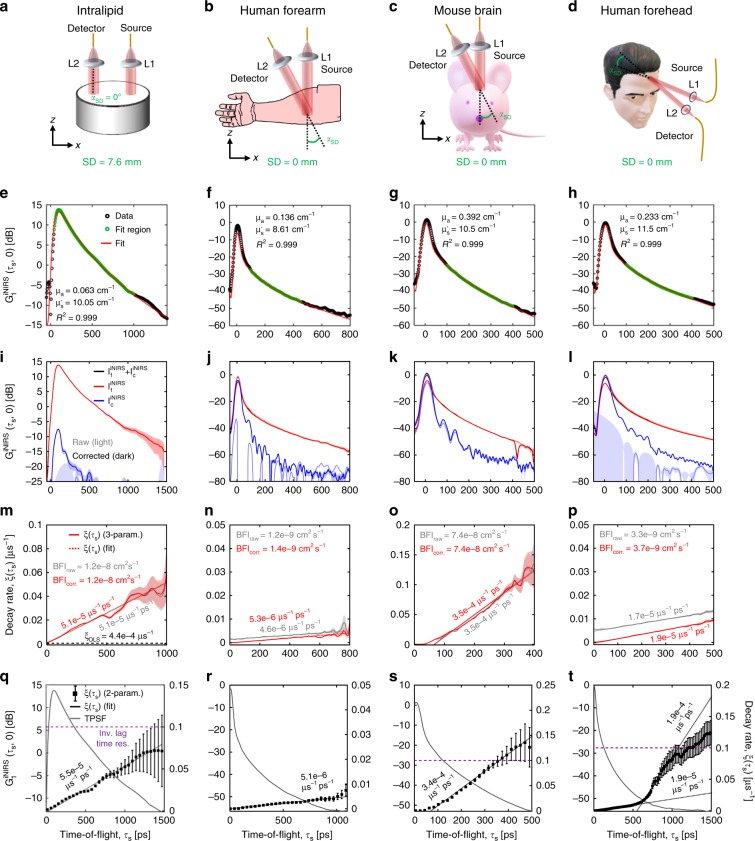
Enabled by motion correction, iNIRS quantifies TOF-resolved dynamics in human tissues. **a**–**d** Non-contact illumination and detection geometry for phantom, mouse, and human measurements. iNIRS, like TD-NIRS, measures temporal point spread functions (TPSFs) which can be fitted with diffusion theory to extract optical properties (absorption and reduced scattering coefficients, ${\upmu}_{\mathrm{a}}$and ${\upmu}_{s}^\prime$, respectively). **e**–**h** Exemplary TPSFs, fitting regions, diffusion theory fits, and extracted optical properties for each respective medium are shown. TOF-resolved iNIRS field autocorrelations can be fitted with a 3-parameter model (two amplitudes, one decay rate), Eq. (7), to yield amplitude distributions (**i**–**l**) and decay rates (**m**–**p**). In vivo, the phase correction procedure (Fig. 2) enhances the static component, likely scattering from extravascular tissue, and reduces the dynamic component decay rate at early TOFs, improving overall agreement with DWS theory. Importantly, the DWS fits (dashed lines) intercept closer to zero TOF: −9.8 ps (raw) to −8.8 ps (corrected) in Intralipid, −280.6 ps (raw) to 30.5 ps (corrected) in the forearm, 59.7 ps (raw) to 57.8 ps (corrected) in the mouse brain, −287.9 ps (raw) to 16.1 ps (corrected) in the human forehead. **q**–**t** TOF-dependent averaging enables measurements at longer TOFs; the inverse of the iNIRS lag time resolution (10 μs) is shown as a dotted violet line. All shaded regions and error bars represent 95% confidence intervals.

iNIRS was performed in an Intralipid phantom (μ_*s*_′ = 10 cm^−1^, μ_*a*_ ~ 0.045 cm^−1^), human forearm, nude mouse head, and human forehead (Fig. 3a–d). TPSFs enabled estimation of optical properties (Fig. 3e–h). After motion correction, an exponential model (Eq. (7)) consisting of a static and dynamic term was fit to TOF-resolved autocorrelations. This model is theoretically well justified, approaching dynamic light scattering (DLS) theory (which is valid for a single backscattering angle) and DWS theory (which is valid at early lag times and late TOFs) for Brownian motion (Supplementary Note 3). The static term accounts for static scattering paths (Supplementary Fig. 3) that decorrelate on time scales longer than the fitting window of 10 ms used for all data. For the Intralipid reflectance measurement, where all particles are dynamic (Fig. 3i), the static component is insignificant (blue). For tissue, (Fig. 3j–l), motion correction reveals a strong static component (blue), which likely describes light scattered only from extravascular tissue. Slopes of the autocorrelation decay rate versus TOF (Fig. 3n, p) are increased by motion correction in human tissues, but remain virtually identical for Intralipid (Fig. 3m), which has no physiological motion, and the mouse head (Fig. 3o), which was stereotactically stabilized. Critically, upon phase correction, fits intercepted the TOF axis much closer to τ_*s*_ = 0 (Eq. (11)), as predicted from DWS theory. BFI values, quantified based on Eq. (11) with estimated reduced scattering coefficients, are included (Fig. 3m–p). Finally, to improve observations at late TOFs we employed a TOF-dependent averaging strategy: at each TOF, τ_*s*_, ${{G}}_1^{{\mathrm{iNIRS}}}$ was averaged across a 20% window (e.g. for τ_*s*_ = 1000 ps, ${{G}}_1^{{\mathrm{iNIRS}}}$ was averaged from 900–1100 ps). Subsequently, resulting autocorrelations were fitted with a 2-parameter model (Eq. (9)), a reasonable approximation at late TOFs (see Supplementary Note 3), to determine decay rates (Fig. 3q–t). Interestingly, human head measurements (Fig. 3t) exhibited two decay stages, with a lower slope at early TOFs, consistent with extracerebral (e.g. scalp and skull) blood flow^31^, transitioning to a higher slope at later TOFs, consistent with higher CBF.

### Bi-exponential field autocorrelations at early to intermediate TOFs

**Fig. 4:**
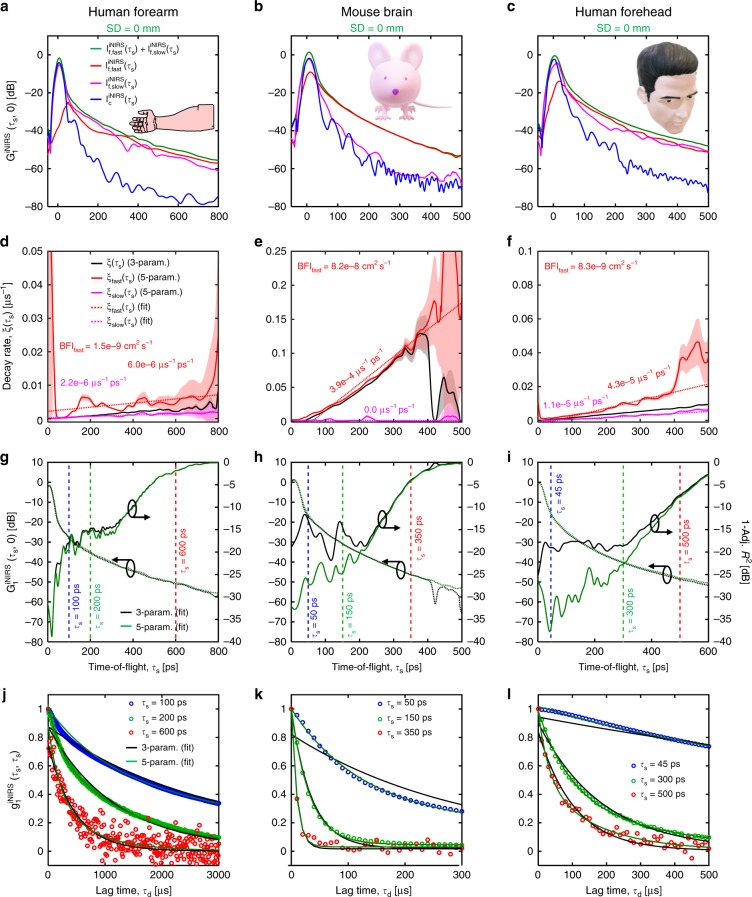
Bi-exponential model is required to fully describe in vivo measurements. **a**–**c** Fitting TOF-resolved field autocorrelations with an empirical bi-exponential 5-parameter model (3 amplitudes, 2 decay rates) reveals the presence of a slow dynamic component. **d**–**f** Corresponding TOF-resolved decay rates, and quantitative blood flow indices for the fast dynamic component (BFI_fast_). Shaded regions represent 95% confidence intervals. **g**–**i** Adjusted *R*^2^ values support the need for the 5-parameter model to accurately describe iNIRS measurements up to intermediate times of flight of 200–400 ps, depending on the tissue, beyond which a 3-parameter model suffices (two amplitudes, one decay rate). **j**–**l** Exemplary field autocorrelations for selected times of flight confirm the need for the 5-parameter model up to intermediate TOFs. Note that with more averaging, a 5-parameter model was needed in the human forehead at even later TOFs (Supplementary Fig. 4).

Careful observation revealed systematic discrepancies between mono-exponential fits and in vivo data, up to TOFs of a few hundred picoseconds. To empirically describe data more comprehensively across time lags, a 5-parameter fit was developed (Eq. (8)). The adjusted *R*^2^, which penalizes overfitting, was determined for both the mono-exponential (3-parameter) and bi-exponential (5-parameter) fits. Besides improving the adjusted *R*^2^ at intermediate TOFs, (Fig. 4g–i) the empirical 5-parameter fit (three amplitudes, two decay rates) eliminated systematic deviations that plagued the 3-parameter fit (two amplitudes, one decay rate), which is founded in DLS and DWS theory.

Based on the empirical 5-parameter fit, TOF-resolved amplitudes were constructed for the static, slow, and fast components and compared across tissues (Fig. 4a–c). First, the fast component dominated at long TOFs and its decay rate increased with TOF. Second, the static component dominated at short TOFs and null SD separation, but rapidly attenuated with increasing TOF in all tissues. Third, a slow component, with a TOF distribution intermediate between the static and fast components, was required to accurately describe in vivo data. The amplitude and decay rate (inverse time constant) of the slow component relative to the fast component were sample dependent. The slow component was notably absent in Intralipid, with a much lower scattering anisotropy than RBCs (*g*_IL_ ~ 0.6^32^ versus *g*_RBC_ ~ 0.975^33^).

**Fig. 5:**
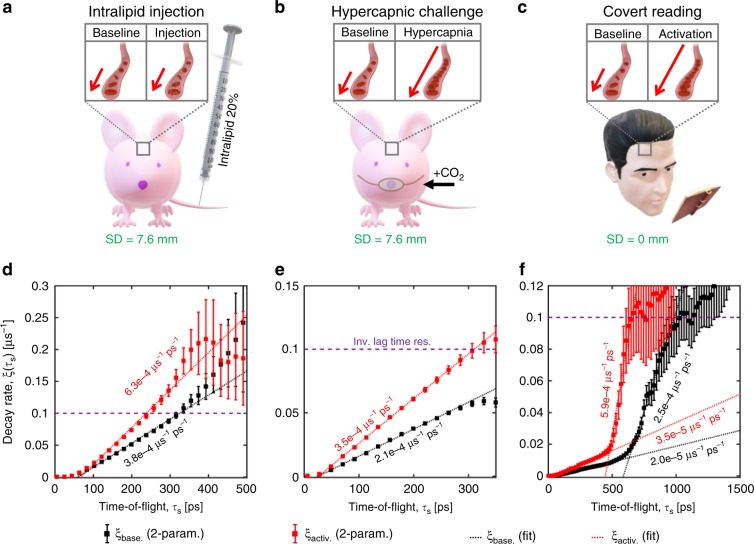
Time-of-flight (TOF) resolved field dynamics during physiological manipulations. TOF-resolved field dynamics were monitored by iNIRS during three distinct physiological manipulations: tail vein injection of highly scattering Intralipid-20% (**a**) and 5% hypercapnic challenge (**b**) in mice, and a covert reading task for prefrontal cortex activation in a human subject (**c**). Decay rates versus TOF, with application of TOF-dependent averaging, reveal changes during all manipulations (**d**–**f**). The extracted blood flow indices are 8.3 × 10^−8^ cm^2^ s^−1^ and 1.4 × 10^−7^ cm^2^ s^−1^ at baseline and after Intralipid injection, respectively, in the mouse brain; 4.7 × 10^−8^ cm^2^ s^−1^ and 8 × 10^−8^ cm^2^ s^−1^ during normocapnia and hypercapnia, respectively, in the mouse brain; and 3.8 × 10^−9^ cm^2^ s^−1^ and 6.7 × 10^−9^ cm^2^ s^−1^ at baseline and during activation, respectively, for the early phase in the human forehead. In the human forehead, the slope of the late phase increases by 140% during activation (**f**). A Monte Carlo simulation, based on a two-layer model with realistic blood flow indices, agrees with experimental findings (Supplementary Fig. 5b). Error bars represent 95% confidence intervals.

Next, we examined changes during various manipulations with TOF-dependent averaging. For separate nude mouse head measurements, we injected 0.1 mL of Intralipid-20% intravenously, which increases the number of dynamic scattering events and momentum transfer (Fig. 5a, d), and performed a standard hypercapnic challenge, which increases cerebral blood flow (Fig. 5b, e). Both manipulations increased the slope of the decay rate versus TOF. For human forehead measurements (prefrontal cortex activation), the subject covertly read a paragraph of unfamiliar text after a 10-min rest period (eyes closed).

The slopes of both of the aforementioned stages increased during activation, with the late slope increasing more than the early slope (Fig. 5c, f). These distinct decay stages are plausible based on known differences in blood flow dynamics in superficial (scalp and skull) and brain tissue^31^. Importantly, the fast dynamic component increased during all manipulations (Supplementary Fig. 6), while the behavior of the slow component was unclear.

To interrogate whether the slow component was intravascular in origin, we then performed simulations based on a time-tested physical model^11^ (Supplementary Fig. 7). First, by eliminating paths with any large angle dynamic scattering events in simulation, we found that the long autocorrelation tails are clearly associated with light paths for which intravascular dynamic scattering events all result in a small deflection angle (Supplementary Fig. 8). The probability of such paths, and hence the shape of the tails, depends on the scattering anisotropy (Supplementary Fig. 9) and even on the shape of the scattering phase function (Supplementary Fig. 10). Due to low blood volume in tissue and high scattering anisotropy of RBCs^33^ (g_RBC_ = 0.975), such paths remain probable up to intermediate TOFs (Supplementary Fig. 11). Second, inclusion of advection and diffusion in a hybrid model did not alter the bi-exponential character of the autocorrelation compared to a pure diffusion (unordered Brownian motion) model, but did change the decay rates of each component and caused the slow component to attenuate more rapidly with TOF (Supplementary Fig. 12). Thus, while the presence of long autocorrelation tails is due to small angle dynamic scattering, the amplitude of the tails also depends on sample dynamics (diffusive versus directional). A hybrid model incorporating advection and Brownian motion (Supplementary Fig. 12) predicted qualitative features of our TOF-resolved autocorrelations. Third, the inclusion of early ballistic displacement affected the short time lag behavior, changed the decay rates of each component, and caused the slow component to attenuate more rapidly with TOF (Supplementary Fig. 12). In summary, our simulations and experiments consistently supported that the presence of a fast and slow component (i.e. the need for a bi-exponential fit) at early TOFs was associated with small angle intravascular forward scattering, though the slow component amplitude was not related in a simple way to the fraction of forward scattering events and sample dynamics. Finally, as discussed further below, bi-exponential autocorrelations at later TOFs in the human forehead required a completely different explanation.

### Deconstructing DWS/DCS

**Fig. 6:**
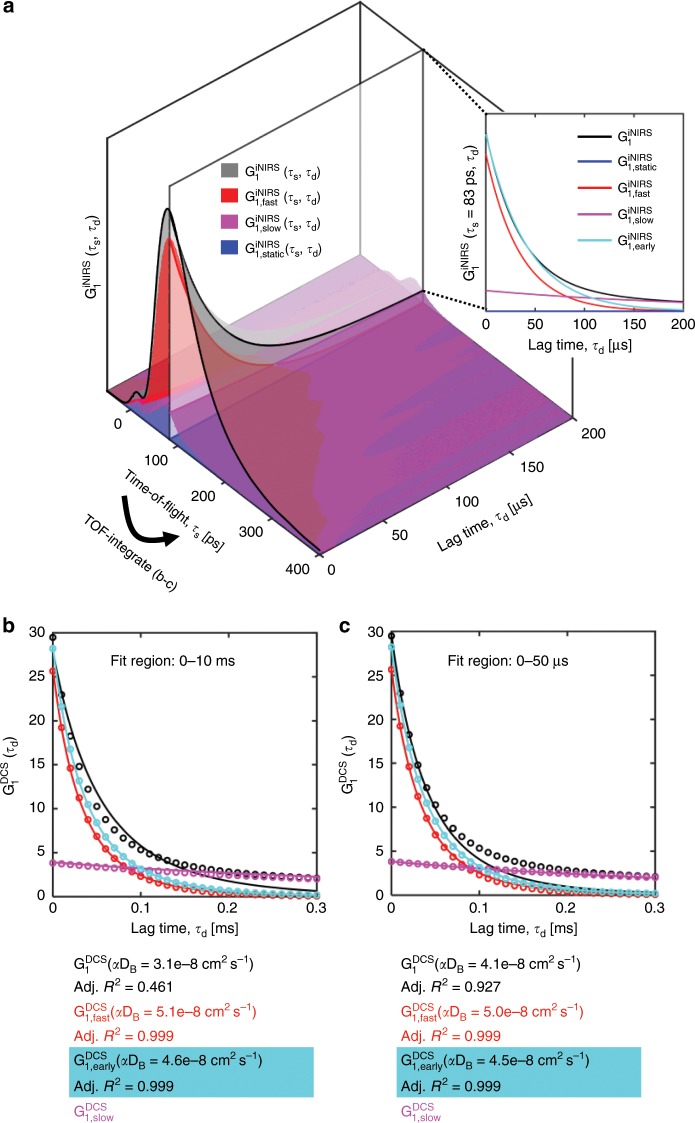
Deconstruction of DWS/DCS with iNIRS. **a** in vivo iNIRS TOF-resolved field autocorrelations, or individual components thereof, can be integrated in TOF to generate DWS/DCS-like autocorrelations. For TOF-integrated DWS/DCS-like autocorrelations, ${{G}}_1^{{\mathrm{DCS}}}$, fitting the entire autocorrelation with DWS/DCS theory results in a poor fit (**b**). Fitting early time lags, where the cumulant approximation is valid, results in a considerably higher adjusted *R*^2^ value, but the fit diverges from the data at large time lags (**c**). On the other hand, integrating just the iNIRS fast component (red) or the iNIRS early time lag single exponential fit (cyan), yields autocorrelations in better agreement with DWS/DCS theory across time lags and adjusted *R*^2^ approaches 1 regardless of fit region (**b**–**c**).

**Fig. 7:**
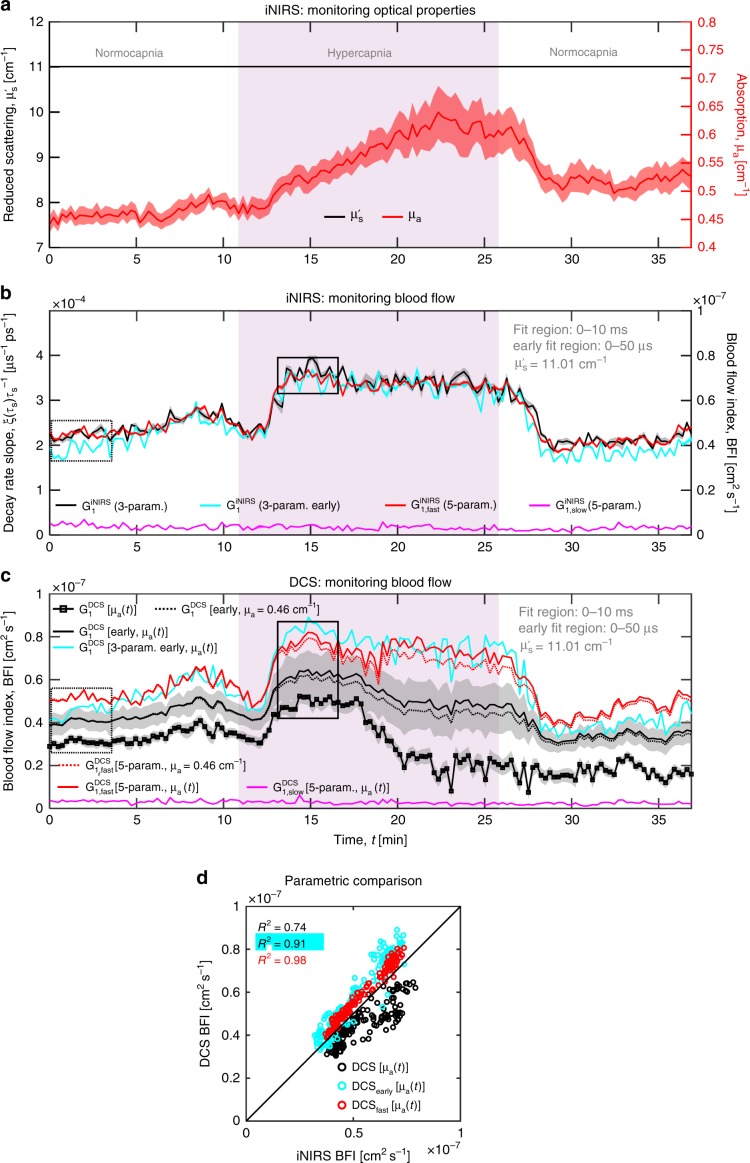
iNIRS deconstructs DWS/DCS during a hypercapnic challenge in the mouse brain. By accounting for absorption changes (**a**), conveniently also measured by iNIRS, and fitting early time lags in DWS/DCS, agreement of DWS/DCS blood flow indices with iNIRS is improved (**b**–**c**). However, even when such best practices are followed in DWS/DCS (black in **d**, the accuracy of DWS/DCS can still be significantly improved either by integrating just the iNIRS fast component (red in **d**) or the iNIRS early time lag single exponential fit (cyan in **d**). This is likely because the slow component (magenta), though intravascular in origin, is not described by conventional DWS/DCS theory (Supplementary Fig. 6).

We next investigated the contributions of the empirical iNIRS slow components to classical TOF-integrated DWS/DCS. To contend with the long autocorrelation tails in iNIRS, in addition to 5-parameter iNIRS fitting (approach 1), we also performed 3-parameter early time lag iNIRS fitting (approach 2), which has a more solid theoretical foundation in the cumulant approximation (see Supplementary Note 3). Then, we integrated ${{G}}_1^{{\mathrm{iNIRS}}}$ over TOF (Fig. 1b, Supplementary Note 4), or we integrated the fast component (red) or early lag fits (cyan) individually (Fig. 6a), to ascertain their role in DWS/DCS. First, we found that that blood flow index (BFI = α*D*_*B*_) of DWS/DCS is highly dependent on the fit region. On the other hand, integrating just the iNIRS fast component (red) or iNIRS early lag fits (cyan) alone yielded better agreement with DWS/DCS theory, resulting in the same BFI regardless of fit region (Fig. 6b, c). In addition to field dynamics, iNIRS quantifies both baseline absorption and changes during hypercapnia (Fig. 7a). However, BFI can be recovered in iNIRS without explicit knowledge of absorption (Fig. 7b). On the other hand, absorption must be considered in DWS/DCS. Reassuringly, iNIRS BFI from the fast component decay rate of the 5-parameter fit (approach 1, red in Fig. 7b) agreed with iNIRS BFI from the early time lag fit (approach 2, cyan in Fig. 7b). We expect that fitting early lags in DWS/DCS should more effectively, but perhaps imperfectly, isolate the fast component, which is sensitive to modulations of intravascular scattering dynamics (Supplementary Fig. 6). Indeed, applying “best practices” in DWS/DCS (fitting early time lags^34^ and accounting for absorption^35,36^) achieves only modest agreement with iNIRS (solid black Fig. 7c). Importantly, agreement is improved significantly by applying DWS/DCS theory to the TOF-integrated fast iNIRS component (red in Fig. 7c), or the TOF-integrated early lag iNIRS fit (cyan in Fig. 7c), both approaches which “force” a single exponential autocorrelation decay at all TOFs. As discrepancies are resolved by eliminating the slowly decaying tails, we can pinpoint the slowly decaying tails as a confound. Evidently, even if best practices are followed in DWS/DCS, TOF-resolved iNIRS can still improve quantification at 7.6 mm SD separation.

### Discussion

In this work, we introduce two new dimensions, optical phase and TOF, to improve DOF of human tissues. To achieve non-contact measurements in vivo, we move the source and detector closer^37^, using the large intrinsically static reference to estimate and correct phase drift due to motion, and custom signal processing to achieve the dynamic range needed to isolate diffuse paths (Supplementary Fig. 1). Our methodology reveals fundamental physics of light–tissue interactions, providing informative and direct diffuse optical measurements that decipher classical techniques.

In DWS/DCS, a Brownian motion model fits experimental autocorrelation functions better than a more intuitive random flow model. Though BFI empirically correlates with standard flow measures^38^, the units of BFI (distance^2^/time) remain challenging to interpret. Simulations have suggested that RBC migration in laminar shear flow, describable as a diffusion process^39^, provides a plausible explanation for Monte Carlo simulations^20,40^. However, the applicability of this concept in capillaries with single-file RBC flow, which account for a majority of microvascular volume, remains unclear. In laser Doppler or laser speckle, which do not possess TOF resolution, it is always possible to invoke contributions from longer, multiply scattered paths to explain exponential autocorrelation decays via an advection, or random flow, model^18,19^.

On the other hand, iNIRS can assess TOF-resolved dynamics essentially independently of the TOF distribution of photons. The observation of exponential decays at early time lags and late TOFs in iNIRS autocorrelations (22 ps TOF resolution) provides the most comprehensive experimental support, to date, for a Brownian model of RBC displacement in coherent light scattering, rendering a pure advection model of RBC motion untenable. Nonetheless, our results are not inconsistent with a hybrid model that incorporates advection and Brownian motion. Our simulations showed that a hybrid model does not alter the bi-exponential character of the TOF-resolved field autocorrelations, but does change the relative decay rates and amplitudes of the fast and slow components (Supplementary Fig. 12). This conclusion also held for early ballistic motion prior to the collision time, though some deviations from the bi-exponential fit were evident prior to the collision time. We found that a hybrid model could recapitulate observed features in our experimental autocorrelations, particularly the relative attenuation of the slow component with TOF. Our sampling rate was insufficient to conclusively identify a finite collision time scale (i.e. very early motion resembling advection) to establish Brownian motion in all tissues (Fig. 4j–l). Finally, we caution that just because autocorrelations are well-described by a Brownian motion model, does not per force imply that RBC diffusion is responsible for the autocorrelation decay. Other motion of RBCs, such as tumbling or deformation, not described by existing theories, may also play a role. Along these lines, it is interesting to note that OCT also measures exponential field autocorrelations in ~0.05 picoliter intravascular volumes, in a quasi-backscattering geometry^41,42^. The connection of such findings to those presented here, if any, requires further investigation.

In vascularized tissue, coherent light transport entails a sequence of static scattering events from tissue matrix, punctuated by dynamic scattering events from blood^11^. For a wide range of in vivo measurements, a constant plus bi-exponential decay provides an excellent empirical description of field autocorrelations at all TOFs, excepting minor deviations at very early time lags in some media (Fig. 4). The fast decay component appears to increase with TOF, agrees with DWS theory based on the cumulant approximation at late TOFs in homogenous media, and increases when intravascular momentum transfer is enhanced (Supplementary Fig. 6).

Across tissues, the slow component was needed, at least up to TOFs of 200–400 ps, for accurate in vivo fits over the entire autocorrelation decay. The slow component was absent in Intralipid phantoms (*g*_IL_ = 0.6), and its amplitude was diminished by Intralipid injection in vivo, strongly suggesting an association with RBC scattering anisotropy. Likewise, in our simulations (Supplementary Figs. 8–10), quasi-forward dynamic scattering events were necessary and sufficient for a bi-exponential autocorrelation in a homogenous medium. This is most clearly shown in Supplementary Fig. 8, where eliminating paths with any large angle dynamic scattering events still yielded an excellent approximation of the long autocorrelation tails. Thus, we propose that paths with low momentum transfer at dynamic scattering events lead to the long autocorrelation tails. These paths can persist in TOF due to the low probability of dynamic scattering in tissue (fractional blood volume ~ 1–5%), and high forward scattering of RBCs^43^. In contrast to traditional forward scattered “snake paths”^44^, these slowly decorrelating paths are “dynamical snake paths” in the sense that they are highly forward scattered only by dynamical tissue (Supplementary Fig. 11). Dynamical snake paths can be highly scattered by the surrounding static tissue matrix, and might obey the diffusion approximation for radiative transport. However, dynamical snake paths invalidate the cumulant approximation and the diffusion approximation for correlation transport, which predicts a pure exponential autocorrelation for uniform Brownian motion along a single light path. Dynamical snake paths, while few in number at larger TOFs, can still influence DWS/DCS autocorrelations at long time lags. Finally, we have not provided a rigorous theoretical treatment of the long autocorrelation tails, in part due to its attribution to two distinct physical effects (Table 1), as well as uncertainties about RBC scattering (Supplementary Fig. 10). Such uncertainties can be circumvented by a mono-exponential, early time lag fit, which is theoretically justified in DWS.

**Table 1:** Physical effects leading to iNIRS observations. Based on in vivo iNIRS data and simulations, we ascribe distinct physical effects to salient iNIRS observations. Support for the ascription of physical effects to iNIRS observations is provided in the form of simulation (sim.) and experiment (exp.).

In TOF-resolved iNIRS data, superficial extracerebral blood flow has a clear signature: a slow autocorrelation decay rate at early to intermediate TOFs (e.g. Figure 5f). By comparison, dynamical snake paths create long autocorrelation tails at late time lags and early to intermediate TOFs. Interestingly, superficial layers with low blood flow create slowly decaying tails at long time lags in TOF-integrated DWS/DCS. Thus, it is reasonable to ask how a thick (~1 cm) superficial layer can affect iNIRS data at late time lags and TOFs. In the human head, applying TOF-dependent averaging to reduce noise (Supplementary Fig. 4), we observed long autocorrelation tails, necessitating a bi-exponential fit, even at TOFs of >500 ps. This observation is likely due to light paths sampling both superficial (scalp/skull) and deep (brain) dynamics, which are 6–10 times higher^31,45^. Monte Carlo simulations (Supplementary Note 5), employing a two-layer model with reasonable blood flow indices, reproduced both the biphasic behavior of the decay rate versus TOF, as well as bi-exponential autocorrelations at late TOFs, in agreement with human forehead experiments (Supplementary Fig. 5). Notably, as the Monte Carlo simulation assumed the cumulant approximation, bi-exponential decays were absent at early TOFs, unlike our experimental human forehead data. Additionally, in the mouse head, we observed that removal of the scalp did not eliminate bi-exponential autocorrelations (data not shown). Thus, the scalp is not required for a bi-exponential autocorrelation, and we must attribute the bi-exponential autocorrelation to two distinct physical effects. These findings are summarized succinctly in Table 1.

Can iNIRS decipher classical DWS/DCS in vivo? Standard practice in DWS/DCS is to fit early time lags, in order to select for longer, deeper photon paths^7,34,40^. First, we note that Brownian motion (Δ*r*^2^ ~ τ_*d*_) must dominate advection (Δ*r*^2^ ~ τ_*d*_^2^) at sufficiently short time scales. Thus, as our results support the presence of Brownian motion, to the extent that DWS/DCS succeeds in isolating the earliest time scales, DWS/DCS will observe Brownian motion. Second, our survey of literature indicates that DWS/DCS early time lag fits often deviate from long decay tails^46,47^ at short SD separations. Our deconstruction of DWS/DCS at short SD separations (Fig. 6) clearly connects these tails with the slow component of our bi-exponential iNIRS decay and by extension, dynamical snake paths. Since the cumulant approximation for uniform Brownian motion yields an exponential TOF-resolved autocorrelation, the very presence of a bi-exponential decay implies that the cumulant approximation with uniform Brownian motion is invalid. Without the benefit of TOF resolution, fitting early lags cannot eliminate the influence of the slow phase (Fig. 7d). Our in vivo results suggest that previous estimates of DWS/DCS blood flow error, based on the cumulant approximation and consequent similarity relation, may be underestimated at short SD separation^48^. To ensure accurate DWS/DCS measurements at short SD separations^48^, and in laser Doppler/laser speckle^11,12^, future work should carefully investigate the validity of the cumulant approximation, which depends on anisotropy (Supplementary Fig. 9) and the phase function (Supplementary Fig. 10) for dynamic scattering. For DWS/DCS at 2.5 cm SD separation on the human forehead, the validity of the cumulant approximation may not be a major concern at early time lags; particularly in relation to the more salient issue of extracerebral contamination^30^.

Can iNIRS decipher classical DWS/DCS at longer SD separations? Preliminary iNIRS decay rates in the adult human head exemplify two stages, consistent with lower extracerebral (i.e. scalp and skull) flow probed at early TOFs, and higher CBF probed more at late TOFs. These data invalidate the assumption of a semi-infinite homogenous medium in DWS/DCS, providing the most direct evidence, to date, of long-assumed differences^31^ in scalp and cerebral blood flow index, supporting that iNIRS can distinguish superficial and deep dynamics. This capability will enable more robust monitoring of CBF autoregulation in brain-injured patients^49^ and higher performance brain computer interfaces^50^ with better spatial resolution and brain specificity.

Coherent light transport in vascularized tissue is a complex sequence of static and dynamic scattering events^11^. We present a unified and comprehensive approach to characterize this process in human tissues, employing an interferometric technique to add new dimensions to experimental measurements. With TOF-resolved autocorrelations, we provide evidence of light paths sampling dissimilar dynamics in multilayered tissues. By applying DWS/DCS theory at early time lags, our approach recovers blood flow indices without explicit knowledge of absorption. At late time lags, we also identify distinct signatures of light paths experiencing small momentum transfer at dynamic scattering events (“dynamical snake” paths). These physical insights could lay the groundwork for a more comprehensive theory that includes late time lags to yield even more information about blood flow dynamics. This work demonstrates that TOF-resolved field dynamics can provide more accurate assessments of blood flow with near-infrared light, through direct observation and also by informing our interpretation of classical techniques.

## Methods

### Interferometric near-infrared spectroscopy

iNIRS is a TOF-resolved method for quantifying field dynamics in turbid media^23,25^. By measuring the interference spectrum of light traversing biological tissue and light traveling a reference path, the technique yields a mutual coherence function, Γ_*rs*_(τ_*s*_, *t*_*d*_) between the sample and reference fields, where τ_*s*_ is TOF and *t*_*d*_ is delay time. From a time series in *t*_*d*_, iNIRS provides a TOF-resolved optical field autocorrelation, ${{G}}_1^{{\mathrm{iNIRS}}}$ (τ_*s*_, τ_*d*_),

1 $$G_1^{{\rm{iNIRS}}}(\tau _s,\tau _d) = \langle \Gamma _{rs}^ \ast (\tau _s,t_d)\Gamma _{rs}(\tau _s,t_d + \tau _d)\rangle _{t_d},$$

where τ_*d*_ is time lag and brackets denote expectation or averaging over *t*_*d*_^25^. The iNIRS field autocorrelation is related to intrinsic medium field autocorrelation, *G*_1_(τ_*s*_, τ_*d*_), by a convolution (*) in TOF (τ_*s*_) with the system instrument response function (IRF):

2 $$G_1^{{\rm{iNIRS}}}\left( {\tau _s,\tau _d} \right) = G_1\left( {\tau _s,\tau _d} \right) \ast {\rm{IRF}}\left( {\tau _s} \right).$$

Thus, iNIRS measures TOF-resolved field autocorrelations, with a TOF resolution determined by the IRF width. Importantly, as implied by Eq. (2), iNIRS implicitly measures the TPSF, *I*^iNIRS^(τ_*s*_) = ${{G}}_1^{{\mathrm{iNIRS}}}$(τ_*s*_, 0) (Fig. 1b), which is related to the intrinsic medium photon TOF distribution (DTOF), *I*(τ_*s*_) = *G*_1_(τ_*s*_, 0), by *I*^iNIRS^(τ_*s*_) = *I*(τ_*s*_) ∗ IRF(τ_*s*_). Therefore, while iNIRS is a coherent method, it also provides a TPSF just like incoherent TD-NIRS^23^. By comparison, DWS/DCS assesses, via the Siegert relationship, TOF-integrated field autocorrelations,

3 $$G_1^{{\rm{DCS}}}\left( {\tau _d} \right) = \frac{1}{\beta }\sqrt {G_2^{{\rm{DCS}}}\left( {\tau _d} \right) - \bar I_{{\rm{DCS}}}^2} = {\int_{0}^{\infty}} {G_1\left( {\tau _s,\tau _d} \right){\rm{d}}\tau _s},$$

where β is the coherence factor and *Ī*_DCS_ is the average detected intensity. Compared to DWS/DCS, iNIRS avoids two problematic operations in Eq. (3). First, by measuring the field autocorrelation directly, iNIRS obviates the Siegert relationship (central expression). Second, iNIRS provides new insight by evaluating the integrand of the right expression of Eq. (3), with high TOF resolution, analogous to the way that TD-NIRS provides new insight into CW-NIRS (Fig. 1b)^51^.

### Determination of optical properties

iNIRS provides a TPSF, which can be fitted with the convolution of the system instrument response function (IRF) and the TOF-resolved diffusion approximation, to extract the absorption and reduced scattering coefficients, μ_*a*_ and ${{\upmu}}_{s}^{\prime}$, respectively^23^. At null SD, we chose a fitting region that starts at a lower TOF of 100 ps for all samples, to ensure validity of the diffusion approximation for radiative transport, while the upper TOF was adjusted based on SNR (green in Fig. 3e–h). For the four sets of samples (Intralipid, human forearm, mouse brain, and human forehead), the iNIRS TPSF, chosen fitting region, diffusion theory fit, extracted optical properties, and goodness-of-fit are included in Fig. 3e–h respectively. The procedure was validated with a diluted Intralipid phantom (true ${{\upmu}}_{s}^{\prime}$ ~ 10 cm^−1^, μ_*a*_ ~ 0.045 cm^−1^), measured at SD = 7.6 mm, where iNIRS recovers ${{\upmu}}_{s}^\prime$ to within 0.5%. We compared null SD and SD = 7.6 mm fitting results in the human forearm and the mouse brain, ensuring that optical properties agreed to within 10%.

### Co-registered validation against OCT

The configuration for validating iNIRS estimation of bulk phase shifts is shown in Fig. 2g; iNIRS (855 nm) is combined with a Thorlabs 1320 nm Telesto OCT system with a dichroic beam splitter. We ensured that the OCT spot stayed within the iNIRS beam during acquisition, and the total illumination power, ~20 mW across a spot size of 2 mm 1/*e*^2^ and 1 mW with a limiting aperture of 3.5 mm, for iNIRS and OCT respectively, adheres to ANSI limits. The bulk Doppler phase shift is given by

4 $$\Delta \theta = \frac{{4\pi }}{{\lambda _0}}\left[ {\frac{{\Delta z + \Delta z\cos \left( {\alpha _{{\rm{SD}}}} \right) + \Delta x\sin \left( {\alpha _{{\rm{SD}}}} \right)}}{2}} \right],$$

where ∆*z* and ∆*x* are motion in the axial and lateral directions, respectively, α_SD_ is the angle between iNIRS illumination and detection, and λ_0_ is the center wavelength (855 nm for iNIRS and 1320 nm for OCT). Note that no additional steps were taken to stabilize the forearm.

### Field autocorrelation models

The goal of coherent optical flowmetry is to infer motion of scattering particles from field autocorrelations. Particle motion is characterized by the mean-squared displacement as a function of lag time, <Δ*r*^2^(τ_*d*_)>. For diffusion, <Δ*r*^2^(τ_*d*_)> = 6*D*_*B*_τ_*d*_ where *D*_*B*_ is the effective Brownian diffusion coefficient. For this model, BFI = α*D*_*B*_ has been proposed^8^. For random flow, <Δ*r*^2^(τ_*d*_)> = *v*^2^τ_*d*_^2^, where *v* is the standard deviation of the velocity distribution. For this model, BFI = α*v*^2^ has been proposed^22^. For hydrodynamic diffusion, <Δ*r*^2^(τ_*d*_)> = 6*D*_*B*_{τ_*d*_ − τ_*C*_[1 − exp(−τ_*d*_/τ_*C*_)]}, where *D*_*B*_ is the hydrodynamic diffusion coefficient and τ_*C*_ is the time scale required to establish Brownian motion^21^. For this model, BFI = α*D*_*B*_ has been proposed^22^. A hybrid model including random flow and Brownian motion, where <Δ*r*^2^(τ_*d*_)> = 6*D*_*B*_τ_*d*_ + *v*^2^τ_*d*_^2^, has also been investigated.

Here we adopt the view^11^ that tissue light scattering is a sequence of intravascular *dynamic* and extravascular *static* scattering events (Supplementary Fig. 3). For DLS, or single scattering with a fixed scattering vector (**q**) with magnitude *q* = |**q**|, static scattering fraction η_c_, and dynamic scattering fraction η_f_ = 1− η_c_,

5 $$g_1^{{\rm{DLS}}}\left( {\tau _d} \right) = \underbrace {\eta _c}_{{\rm{static}}\;{\rm{component}}} + \underbrace {\eta _f\exp \left[ { - q^2\left\langle {\Delta r^2\left( {\tau _d} \right)} \right\rangle /6} \right]}_{{\rm{dynamic}}\;{\rm{component}}}.$$

In the diffusing wave spectroscopy (DWS) regime^7^, the normalized TOF-resolved field autocorrelation ${\mathrm{g}}_1^{{\mathrm{DWS}}}$(τ_s_, τ_d_) is given by

6 $$g_1^{{\rm{DWS}}}\left( {\tau _s,\tau _d} \right) = \frac{{G_1\left( {\tau _s,\tau _d} \right)}}{{I\left( {\tau _s} \right)}} = \exp \left[ { - \frac{1}{3}\alpha k^2\left\langle {\Delta r^2\left( {\tau _d} \right)} \right\rangle {\upmu} ^\prime _sc\tau _s/n_r} \right].$$

Here, *L* = *c*τ_*s*_/*n*_*r*_ is the photon path length, *c* is the speed of light in vacuum, *n*_*r*_ is the group refractive index (assumed equal to the phase refractive index), *k* = 2π*n*_*r*_/λ_0_ is the medium wavenumber, and λ_0_ is the free space central wavelength. A probability of dynamic scattering, α, is included in DWS theory to account for the presence of static scatterers^46^. Comparing dynamic components of DLS (Eq. (5)) to DWS (Eq. (6)), we observe that, in both regimes, diffusion yields an exponential decay and random flow yields a Gaussian decay.

iNIRS affords the unique ability to measure TOF-resolved autocorrelations spanning from the DLS to the DWS regimes. We might expect that DLS theory (Eq. (5)) describes the earliest TOFs, particularly for the null SD geometry. For intermediate TOFs, between DLS and DWS regimes, integration over scattering angles and/or scattering events is required, and the models are more complicated (Supplementary Note 3) although the cumulant approximation remains valid at early time lags. Finally, we expect a transition to the DWS regime (Eq. (6)) at long TOFs where the cumulant approximation is valid over the entire autocorrelation decay. Early time lag behavior of TOF-resolved autocorrelation is more directly relatable to particle motion than early time lag behavior of TOF-integrated autocorrelations, which depend on the TOF distribution and optical properties (Eq. (3)).

DWS/DCS provides a TOF-integrated field autocorrelation (Eq. (3)) via the Siegert relationship. If optical properties are assumed (both μ_*a*_ and ${\upmu}_{s}^\prime$), TOF-integrated autocorrelations can be fit with Eq. (3) to yield BFI. In iNIRS, BFI can be determined from TOF-resolved autocorrelations, by fitting ${{g}}_1^{{\mathrm{iNIRS}}}$(τ_*s*_, τ_*d*_) = ${{G}}_1^{{\mathrm{iNIRS}}}$(τ_*s*_, τ_*d*_)/I^iNIRS^(τ_*s*_) with Eq. (6), after assuming ${\upmu}_{s}^\prime$, which can conveniently be determined directly from the iNIRS measurements themselves^23,52^. For instance, for the diffusion model, the blood flow can be calculated as BFI = α*D*_*B*_ = λ_0_^2^ × slope/8π^2^*n*_*r*_${\upmu}_{s}^{\prime}$*c*, given the slope of ξ(τ_*s*_) versus τ_*s*_. The extra τ_*s*_ dimension provided by iNIRS yields a richer data set than DWS/DCS to evaluate candidate BFI models, and does not require knowledge of, or assumptions about, absorption. However, the convolution (TOF-integration) in Eq. (3) may result in a non-zero second cumulant if the TOF resolution is poor, which could possibly confound Gaussian and exponential decays. Hence to accurately distinguish between a diffusion model (exponential decay in τ_*d*_) and a random flow model (Gaussian decay in τ_*d*_), fine TOF resolution, not mere TOF discrimination^14^, is essential. In this study, the iNIRS TOF resolution, δτ_*s*_ = 21.9 ps, corresponding to a path length resolution of 6.6 mm in vacuum, is the highest value reported to date^5,53^.

### Fitting approach

At first glance, to encompass DLS (Eq. (5)) and DWS solutions (Eq. (6)), it is reasonable to fit iNIRS field autocorrelations with the following model^52^:

7 $$G_1^{{\rm{iNIRS}}}\left( {\tau _s,\tau _d} \right) = \underbrace {I_c^{{\rm{iNIRS}}}\left( {\tau _s} \right)}_{{\rm{static}}\;{\rm{component}}} + \underbrace {I_f^{{\rm{iNIRS}}}\left( {\tau _s} \right){\rm{e}}^{ - \xi \left( {\tau _s} \right)\tau _d}}_{{\rm{dynamic}}\;{\rm{component}}}.$$

Here, *I*_*f*_ is the TOF-dependent coefficient accounting for the dynamic component of the field, while *I*_*c*_ accounts for the possible presence of static paths. While Eq. (7) was sufficient to describe Intralipid autocorrelations; in vivo, we empirically found that a 5-parameter fit was needed to accurately describe the intermediate regime between DLS and DWS across species and tissue types:

8 $$G_1^{{\rm{iNIRS}}}\left( {\tau _s,\tau _d} \right) = \underbrace {I_c^{{\rm{iNIRS}}}\left( {\tau _s} \right)}_{{\rm{static}}\;{\rm{component}}} + \underbrace {I_{f,{\rm{slow}}}^{{\rm{iNIRS}}}\left( {\tau _s} \right){\rm{e}}^{ - \xi _{{\rm{slow}}}\left( {\tau _s} \right)\tau _d}}_{{\rm{slow}}\;{\rm{dynamic}}\;{\rm{component}}} + \underbrace {I_{f,{\rm{fast}}}^{{\rm{iNIRS}}}\left( {\tau _s} \right){\rm{e}}^{ - \xi _{{\rm{fast}}}\left( {\tau _s} \right)\tau _d}}_{{\rm{fast}}\;{\rm{dynamic}}\;{\rm{component}}}.$$

Note that while the 3-parameter fit is well-founded in DLS and DWS theory, we provide no theoretical justification for the 5-parameter fit. Though the 5-parameter fit was generally required, in the DWS regime, at long TOFs and early time lags, decays are purely exponential. In such circumstances, to reduce the number of parameters, it is reasonable to use a 2-parameter fit:

9 $$G_1^{{\rm{iNIRS}}}\left( {\tau _s,\tau _d} \right) = \underbrace {I_f^{{\rm{iNIRS}}}\left( {\tau _s} \right){\rm{e}}^{ - \xi \left( {\tau _s} \right)\tau _d}}_{{\rm{dynamic}}\;{\rm{component}}}.$$

Finally, well into the DWS regime, the normalized autocorrelation is described even more succinctly by a 1-parameter fit:

10 $$g_1^{{\rm{iNIRS}}}\left( {\tau _s,\tau _d} \right) = \underbrace {{\mathrm{e}}^{ - \xi \left( {\tau _s} \right)\tau _d}}_{{\mathrm{dynamic}}\;{\mathrm{component}}}.$$

In DWS (Eq. (6)), for Brownian motion, the TOF-resolved decay rate is

11 $${\xi} \left( {\tau _{s}} \right) = \frac{{2k^{2}\alpha D_{B}{{\upmu}} _{s}^{\prime} c({\tau _{s} - \tau _{s,0}})}}{{n_{r}}}.$$

Though DWS for a uniform medium passes through the origin, i.e. τ_*s*,0_ = 0, we allow for a non-zero intercept with the TOF axis. This semi-empirical adjustment to theory is believed to account for in vivo media with a thin superficial layer that has comparatively little blood flow relative to the deeper layers, such as the outer layers of the skin in humans, or the scalp and skull in mice. Applying Eq. (7) and Eq. (11) to the Intralipid phantom, we obtained τ_*s*,0_ = −8.8 ps (equal to zero, up to the TOF resolution) and α*D*_*B*_ = 1.18 × 10^−8^ cm^2^ s^−1^ (Fig. 3m), consistent with reported results^35^.

### Static tissue may violate the Siegert relationship

By measuring optical field autocorrelations directly, we also detect the presence of an intrinsically static component, whose phase shift dynamics correlate with accepted “static” tissue measured by OCT (Fig. 2). At null SD separation, this intrinsically static component corresponds mainly to backscattered or back-reflected light from static tissue, and at larger SD separations, light that scatters multiple times from extravascular tissue, never experiencing phase shifts at dynamic scattering events. As might be expected, there is no measurable static component (Fig. 3i) for a uniformly dynamic medium, Intralipid, in a backscattering geometry. Also, in biological tissue, which comprises stationary and moving scatterers, the static component approximates the IRF in a backscattering geometry, and occurs earlier in TOF than all dynamic components (Fig. 3j–l). It is important to note that if the measurement time were shorter than the decorrelation time of the static component, the Siegert relationship would be violated. However, our fitting procedure correctly extracts dynamic component(s) regardless of the measurement time scale (Supplementary Note 6).

### Experimental setup

The iNIRS light source (Fig. 1a) is an 855 nm distributed feedback (DFB) laser, operating at a 100 kHz sweep rate, including forward and backward sweeps, enabling a lag time (τ_*d*_) resolution of 10 μs. A lag time resolution of 10 μs for an exponential field autocorrelation is equivalent to a lag time resolution of 5 μs for the corresponding exponential intensity autocorrelation. The end-to-end tuning range was 64.7 GHz, enabling a TOF resolution of 21.9 ps (IRF FWHM). This TOF resolution exceeds that of state-of-the-art TD-NIRS systems^53^ as well as TD-DCS^14^. The spectral interference signal from the balanced detector was acquired continuously in blocks of 2.5 s at a 200 MHz sampling rate, along with laser sweep triggers. Forward and backward sweeps were segregated, resampled, reshaped, and Fourier transformed as previously described^26^, forming a complex mutual coherence function time series, Γ_*rs*_(τ_*s*_, *t*_*d*_), at each TOF (τ_*s*_) with 250,000 delay time (*t*_*d*_) points, from which autocorrelations ${\mathrm{G}}_1^{{\mathrm{iNIRS}}}$(τ_*s*_, τ_*d*_) were calculated. All samples were illuminated with 20 mW across a spot size of ~2 mm (1/*e*^2^), strictly adhering to the tissue irradiation limits set by the American National Standard Institute (ANSI). The single mode source and detector fibers are collimated with off-the-shelf adjustable aspheric FC collimators (Thorlabs CFC-2X-B). Mouse experiments were performed with the animal in a stereotaxic frame under 1–1.5% isoflurane in a mixture of air and oxygen. All procedures and protocols involving human subjects research were approved by the UC Davis Institutional Review Board and all procedures involving animal subjects were approved by the UC Davis Institutional Animal Care and Use Committee (IACUC).

Compared to our previous work^26,52^, we reduced the source–detector separation here to collect light from intrinsically static tissue and increase the number of detected photons at all times of flight^37^. In this approach, the large backscattered/few-scattered component at early TOFs can obscure later TOFs if the IRF has sidelobes. In contrast with TD-NIRS, the iNIRS TPSF is obtained by Fourier transforming the measured interference signal, enabling apodization techniques to reduce sidelobes, and in turn, increase dynamic range as described in Supplementary Fig. 1.

The interferometric strategy also obviates photon counting detectors (i.e. PMTs and SPADs) susceptible to afterpulses and/or diffusion tails, which reduce dynamic range and contaminate information at long times of flight^26^. In contrast, iNIRS, without gating, achieves comparable dynamic range (~70 dB) to state-of-the-art TD-NIRS systems^54^, without exhibiting afterpeaks, and maintaining high peak-to-sidelobe ratio (~40 dB)^26^. Finally, our interferometric approach is uniquely suited for operation under ambient light conditions.

### Reporting summary

Further information on research design is available in the Nature Research Reporting Summary linked to this article.

## Supplementary information

Supplementary Information

Reporting Summary

## Data Availability

All data supporting the findings reported in this study are available in the article itself and Supplementary Information document. Further inquiries regarding the reproduction of published findings are available from the corresponding author upon reasonable request.

## Source data

Source Data

## Code availability

All algorithms and methods required to reproduce reported findings in the article are described in the article itself and the Supplementary Information. Code can be made available upon reasonable request from the corresponding author.
