# Supplementary material for: Time-of-flight resolved light field fluctuations reveal deep human tissue physiology
Source: Nat Commun. 2020 Jan 20;11:391. doi: 10.1038/s41467-019-14228-5 (PMC6971031; doi:10.1038/s41467-019-14228-5)
Supplement: Supplementary file 1 — Supplementary Information [file 41467_2019_14228_MOESM1_ESM.pdf]

Supplementary Information

Time-of-flight resolved light field fluctuations reveal deep human tissue physiology

Kholiqov et al.

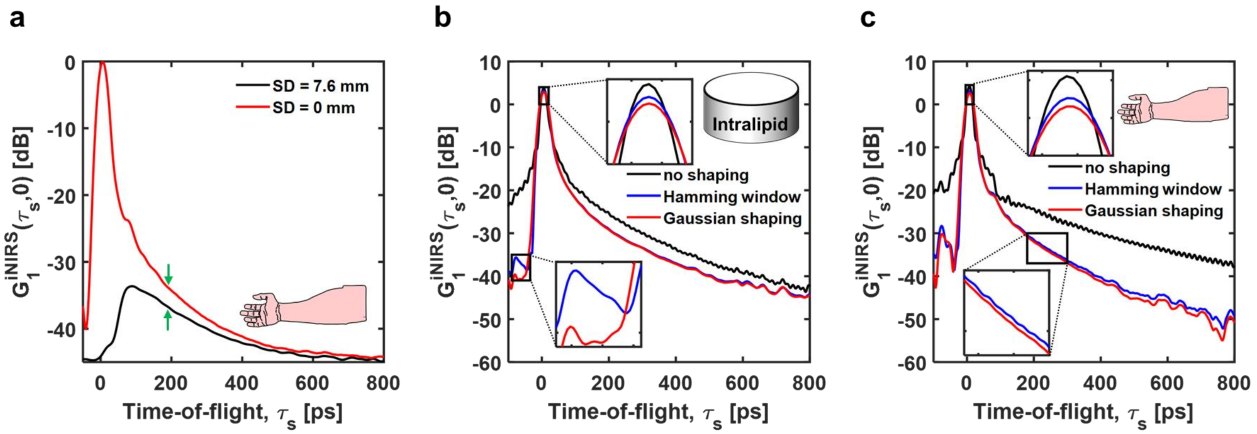

Supplementary Figure 1. Spectral shaping and null-SD detection. Two synergistic technical advances enable iNIRS of human tissues in vivo: null source-detector (SD) separation increases photon counts and provides an intrinsically static backscattered component from which to estimate motion, while Gaussian spectrum shaping enhances dynamic range. **a**, Null SD compared to SD=7.6 mm used previously[41,42] the null SD measurement increases the number of collected photons at every time-of-flight (green arrows). **b**, In Intralipid, Gaussian shaping provides higher dynamic range, as illustrated by the reduction in sidelobes, with a minor (< 0.5 dB) sensitivity loss and negligible degradation of time-of-flight resolution (< 1 ps). **c**, In addition, *in vivo*, Gaussian shaping also mitigates sidelobes resulting from the backscattered static component.

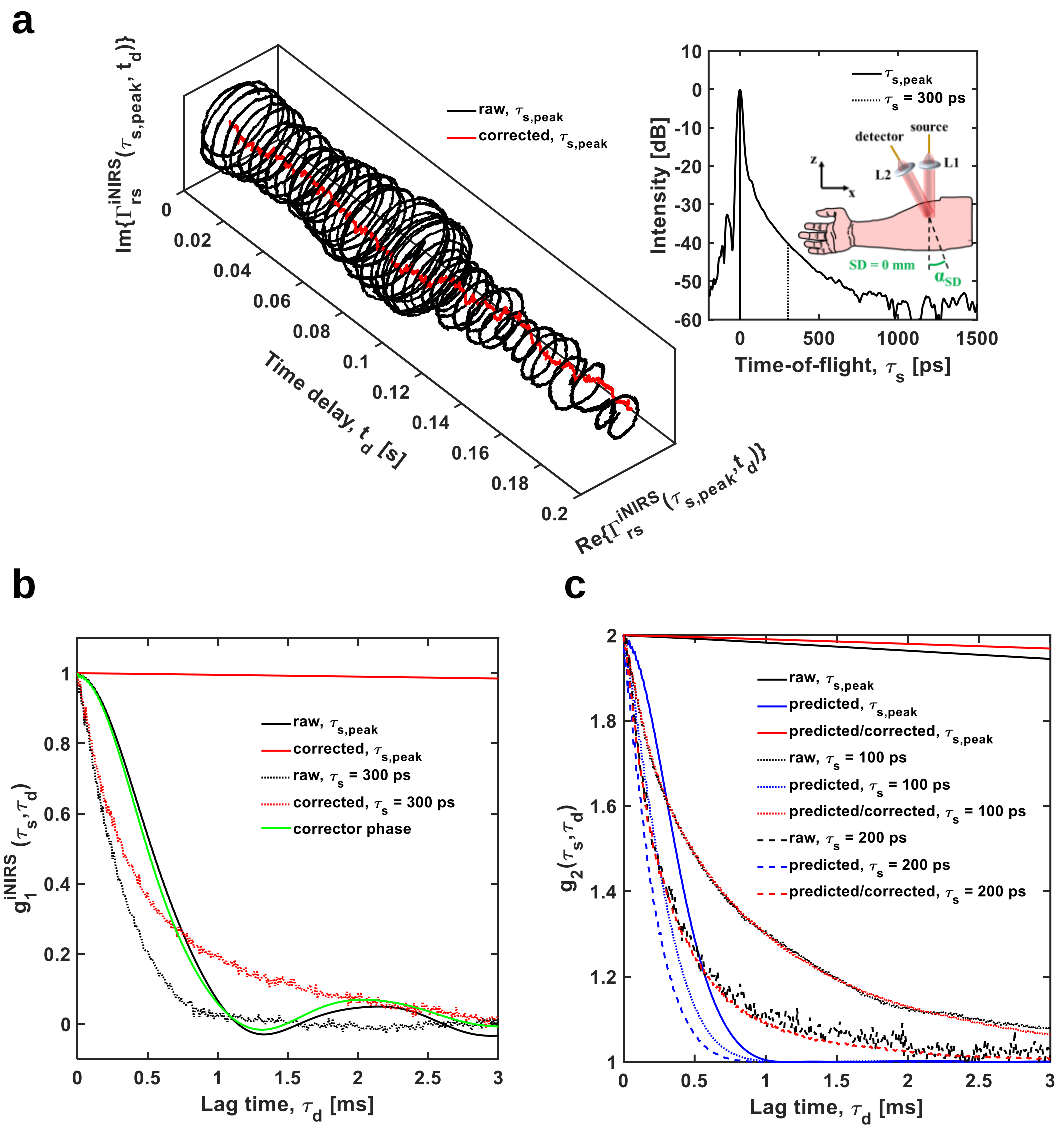

Supplementary Figure 2. **a**, Application of the motion correction method (Figure 2) in the human forearm eliminates phase drift over time delay, as shown by comparing the raw (black) and corrected (red) complex fields. **b**, Correction significantly impacts early TOF autocorrelations (solid black and red), while later TOF autocorrelations (dotted black and red) are less impacted due to the more rapid intrinsic decorrelation. **c**, Exemplary autocorrelations in the human forearm show that bulk phase shifts invalidate the modified or heterodyne Siegert relationship, which relates the intensity autocorrelation (g_2) and field autocorrelation (g_1). Our phase correction method brings the predicted intensity autocorrelation (i.e. g_2 predicted from g_1) into agreement with the directly estimated intensity autocorrelation g_2, restoring the validity of the modified Siegert relationship.

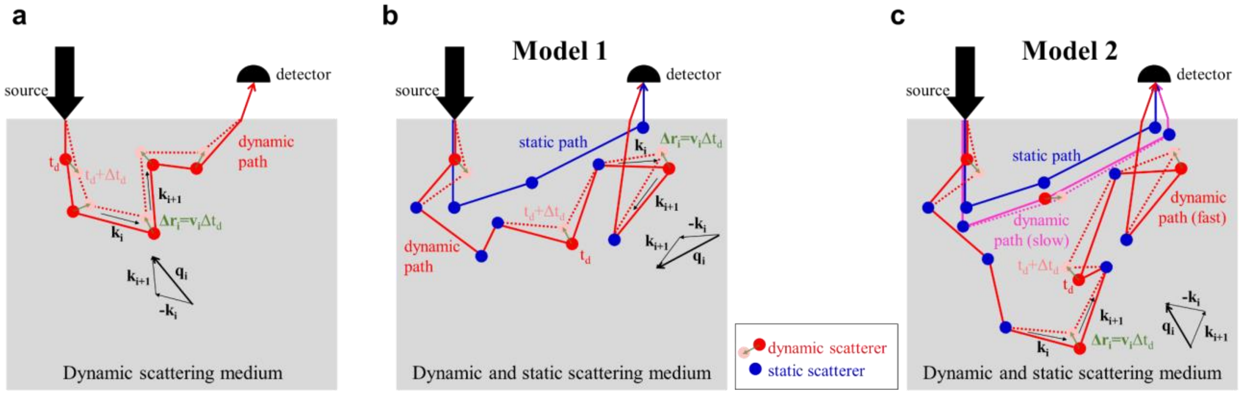

Supplementary Figure 3. Static and dynamic scattering models. **a**, In a sample with intrinsic random dynamics in a reflection mode geometry, momentum transfer must be imparted to the incident light for it to reach from the source to the detector. Any light path with momentum transfer (q=|**q**|) should experience random phase shifts and decorrelate, leading to a purely dynamic TPSF. In a purely backscattering geometry, the slowest expected field autocorrelation decay rate contribution is q^2/D_B, where D_B is the effective Brownian diffusion coefficient and q=2k is the momentum transfer for backscattering. **b**, In a biological tissue, we envision a collection of static, extravascular and dynamic, intravascular scatterers. Only dynamic scattering events with momentum transfer are eligible to contribute to autocorrelation decays (red path). In such a medium, paths with purely static scattering events are possible (blue path), although they become less likely with increasing path lengths. A biexponential decay (model 2) accounts for the slow phase of the autocorrelation decay caused by paths with just quasi-forward dynamic scattering events, with consequent low momentum transfer and small phase shifts (magenta).

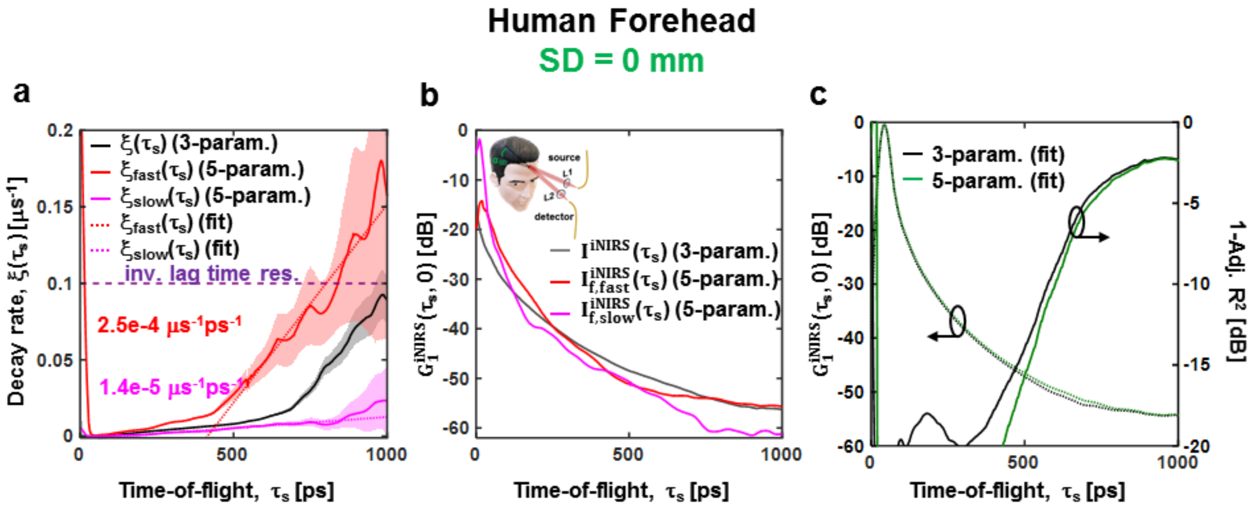

Supplementary Figure 4. Time-of-flight- (TOF-) dependent averaging in the human forehead. Judging from a comparison of the adjusted R^2 values (**c**) for the 3-parameter and 5-parameter fits, the human forehead requires a 5-parameter fit to fully describe optical field dynamics well beyond 500 ps (**a**-**b**). This may be due to light paths sampling scalp and skull, with slower RBC dynamics, as well as the brain, with faster RBC dynamics.

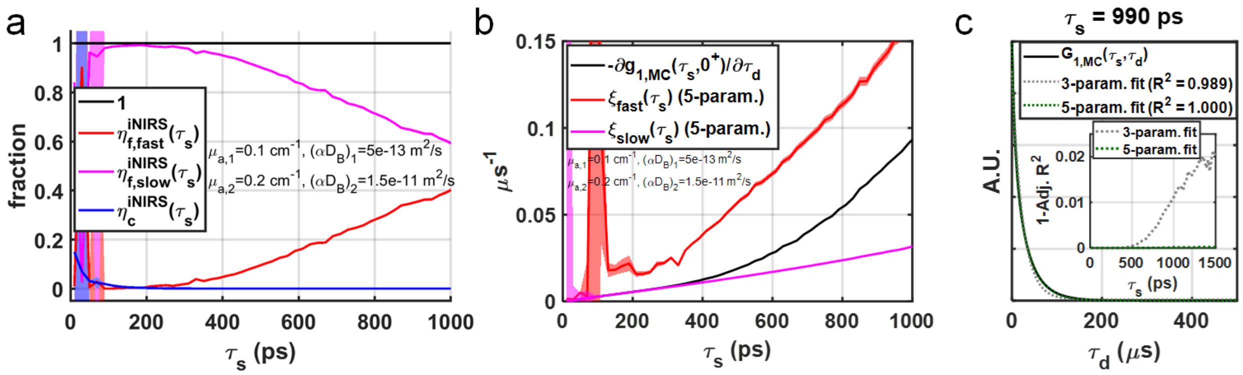

Supplementary Figure 5. Cerebral and extracerebral mixing can lead to biexponential TOF-resolved autocorrelation decays. **a-b**, Fitting of autocorrelations from Monte Carlo (MC) simulation with a 5-parameter, bi-exponential, model used for experimental data. Judging from a comparison of the adjusted R^2 values for the 3-parameter and 5-parameter fits (**c**), a simpler 3-parameter fit suffices up to τ_s=500 ps, but after this time, when scalp and cerebral blood flow are both sampled by photon paths, a 5-parameter fit is required. Thus, mixed sampling of cerebral and extracerebral blood dynamics can lead to a bi-exponential decay at late TOFs. However, an alternative explanation must be invoked to explain bi-exponential decays at early TOFs, which are observed experimentally.

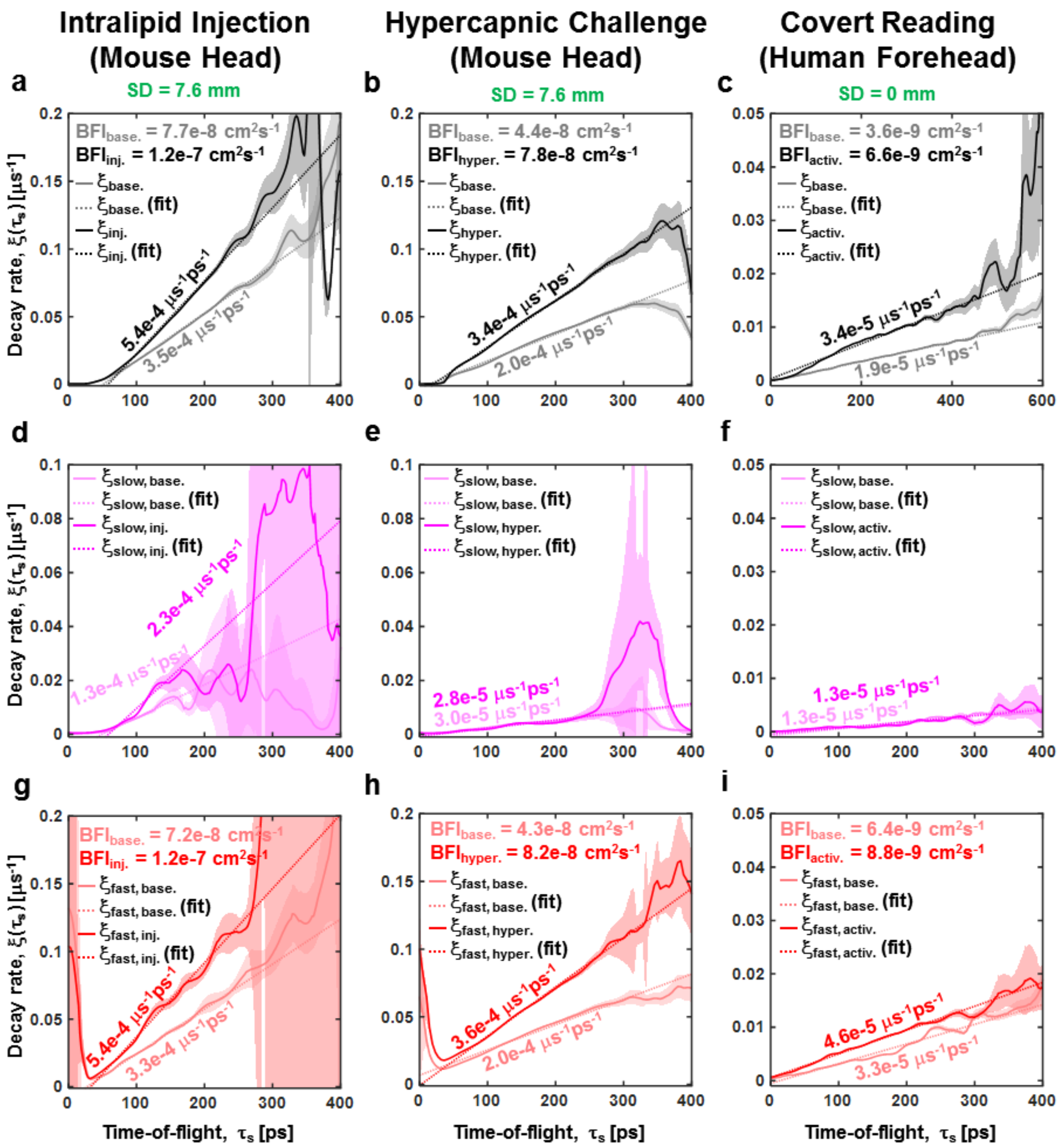

Supplementary Figure 6. Fast and slow dynamic components during physiological manipulations. As expected, for all physiological manipulation scenarios outlined in Figure 5, three parameter fits, Eq. (7) in the main text, exhibit increase in dynamics with Intralipid injection and activation (**a**-**c**). Furthermore, 5-parameter fits, Eq. (8) in the main text, reveal that the fast components (**g**-**i**) increase with Intralipid injection and activation, while the behavior of the slow component (**d**-**f**) is less clear. All shaded regions are 95% confidence intervals.

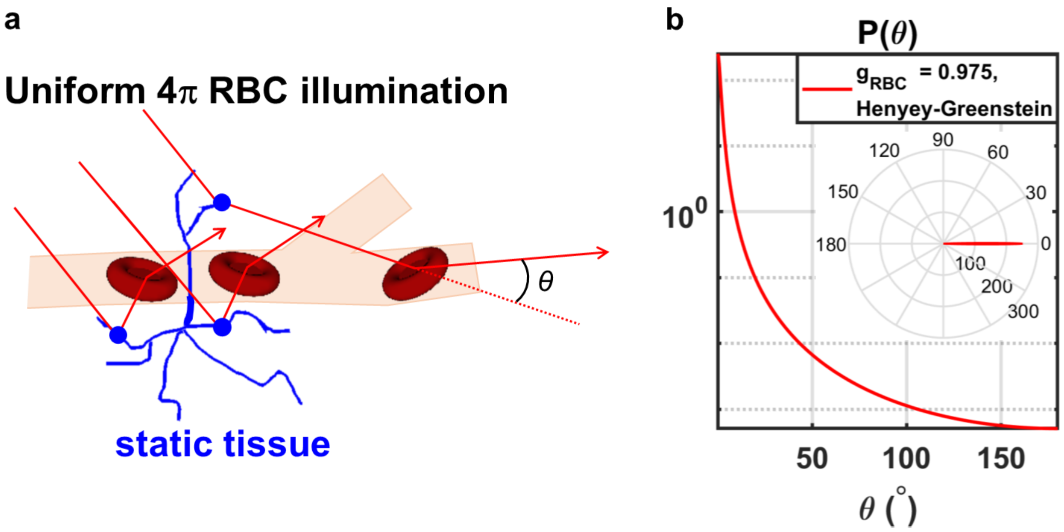

Supplementary Figure 7. **Random illumination of RBCs in biological tissue.** **a**, Scattering from the static tissue matrix is assumed to randomize incident light direction for dynamic scattering events at RBCs. **b**, An azimuthally symmetric phase function P(θ) is assumed for RBC dynamic scattering where θ is the elevation angle.

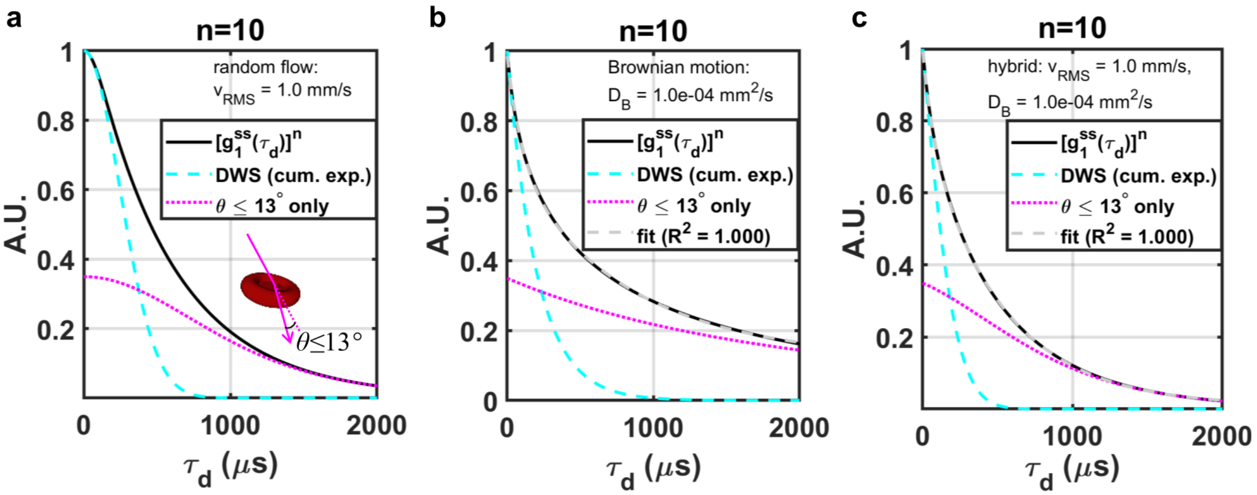

Supplementary Figure 8. Long tails of field autocorrelations require small-angle scattering from dynamic RBCs. Field autocorrelations were simulated for n=10 dynamic RBC scattering events with three models of RBC motion: **a**, random flow or advection, **b**, diffusive or Brownian motion, and (**c**) hybrid motion. For the latter two models (**b**-**c**), our bi-exponential, 5-parameter fit provided an excellent description of the simulated data (gray dashed line, R^2=1.000). The field autocorrelation was also simulated by excluding paths with any dynamic scattering events with a polar angle θ greater than 13° (magenta dotted line). Though constituting only 35% of the paths, θ ≤ 13° paths still dominate at late autocorrelation lags. The DWS/DCS cumulant approximation is also shown for comparison (cyan dashed line).

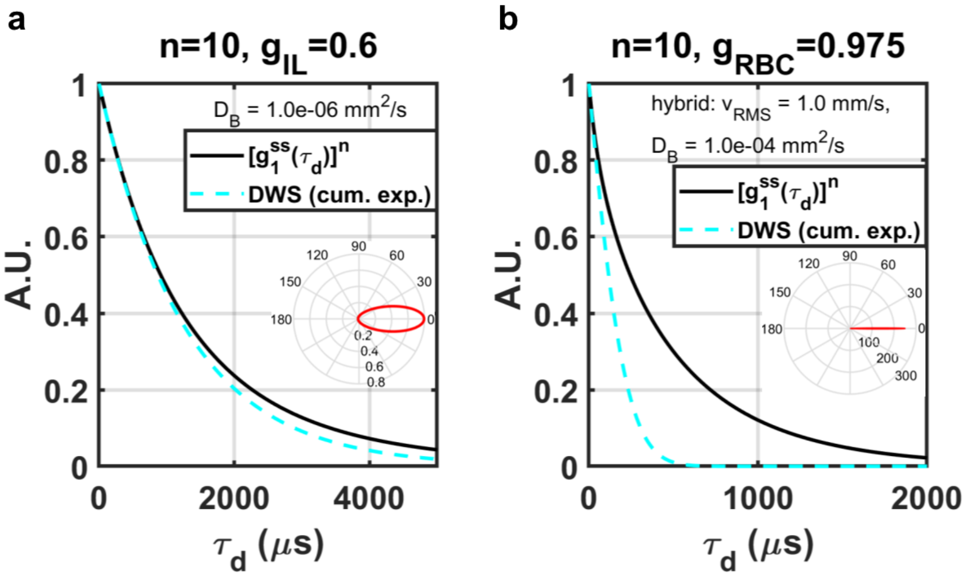

Supplementary Figure 9. The validity of the DWS/DCS cumulant approximation depends on the anisotropy of the scattering phase function P(θ). Autocorrelations were simulated for dilute Intralipid-20% (g_{IL}=0.6) with a Brownian motion model (**a**) and RBCs (g_{RBC}=0.975) with a hybrid motion model (**b**). While the cumulant approximation provides an excellent description of field autocorrelation from the low g medium after just n=10 dynamic scattering events (**a**), the cumulant approximation is much poorer in the high g medium after the same number of scattering events. The cumulant approximation is expected to improve at earlier time lags, and after more dynamic scattering events.

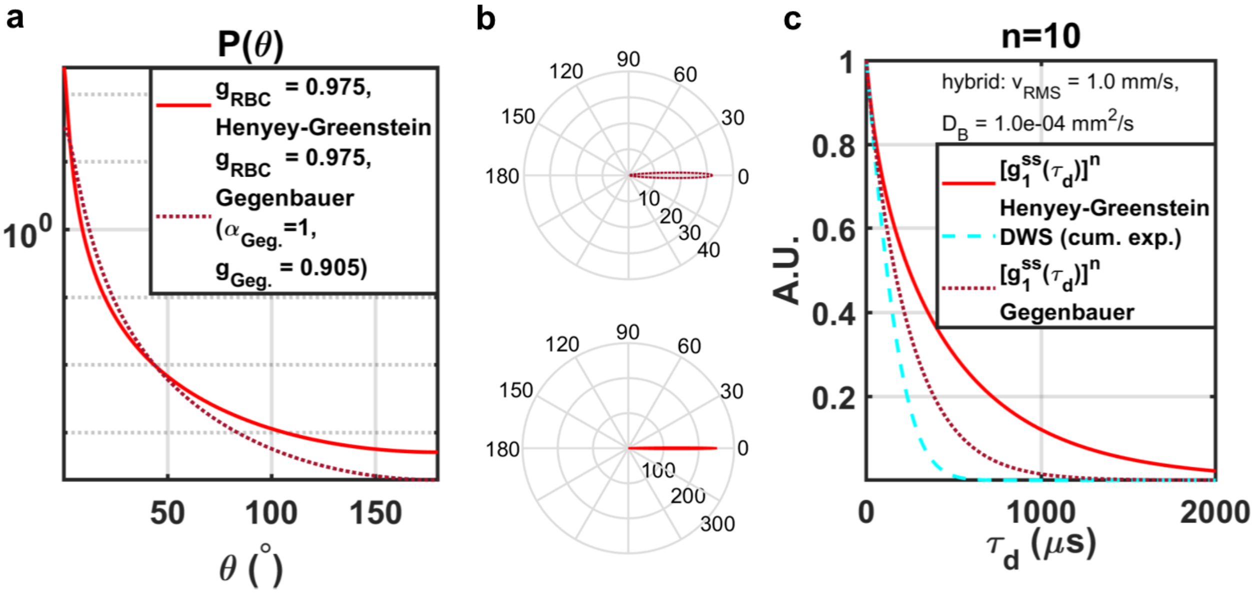

Supplementary Figure 10. The validity of the DWS/DCS cumulant approximation depends on the shape of the scattering phase function P(θ). Two different scattering phase functions (Henyey-Greenstein and Gegenbauer with $\alpha_{Geg.} = 1$) with identical scattering anisotropies are shown (**a**-**b**). **c**, The cumulant approximation (cyan dashed line) provided a better autocorrelation estimate for the Gegenbauer phase function after n=10 dynamic RBC scattering events. The cumulant approximation is expected to improve at earlier time lags, and after more dynamic scattering events.

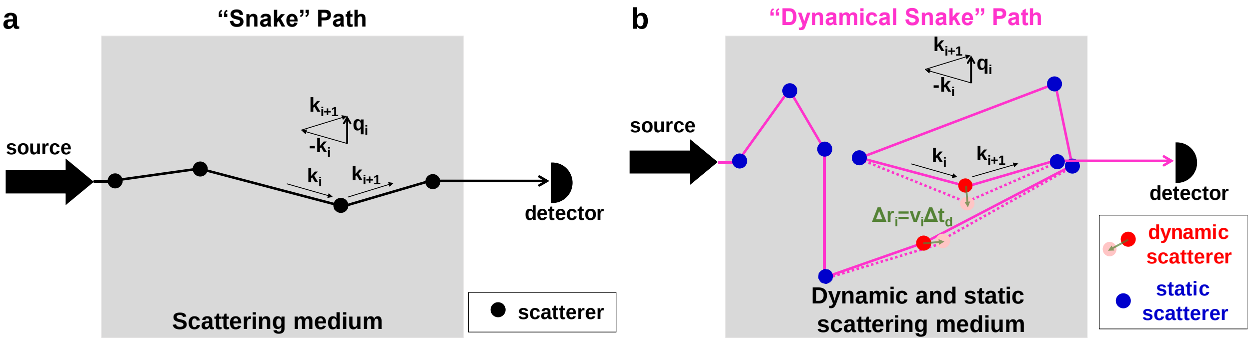

Supplementary Figure 11. “Snake” versus “dynamical snake” paths. The slow dynamic component can be attributed to “dynamical snake” path. While classical “snake paths” (**a**) that experience forward scattering, “dynamical snake” paths (**b**) experience solely forward scattering paths from dynamic scatterers (e.g. RBCs). Dynamical snake paths lead to deviations from the cumulant approximation at larger time lags (Supplementary Figure 8).

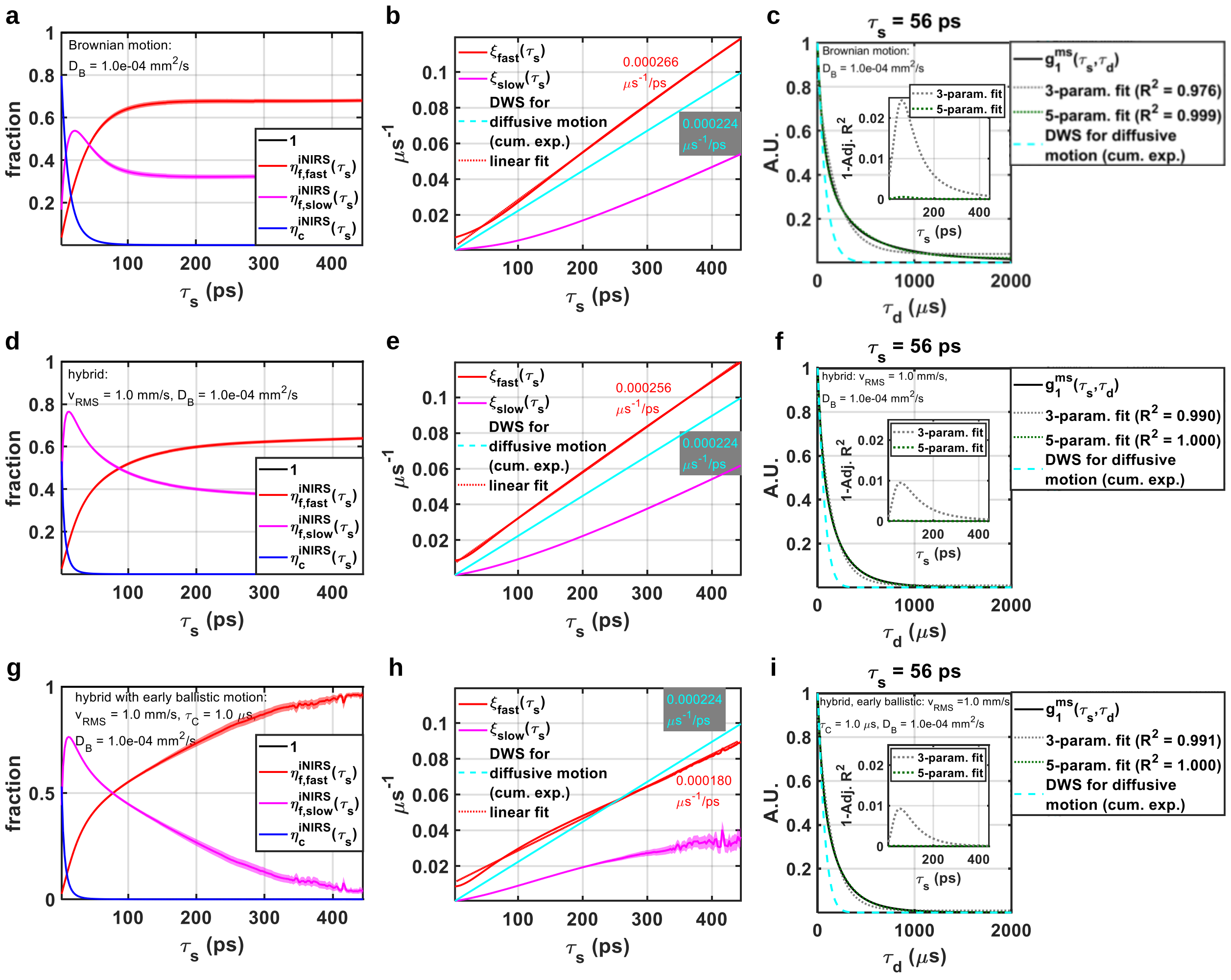

Supplementary Figure 12. Simulations based on Supplementary Eq. (7) for different models of RBC motion in a homogeneous medium. While the bi-exponential fit provides a good description for all models, including Brownian motion (**a-c**), hybrid Brownian motion and random flow (**d-f**), and hybrid with early ballistic motion (**g-i**), the relative amplitudes and decay rates of the slow and fast components differ. Generally, the fast component agrees better with the DWS prediction for a diffusion model (cyan dotted line). Judging from a comparison of the adjusted R^2 values for the 3-parameter and 5-parameter fits, two exponentials are required at early TOFs of 200 ps or less, but a single exponential suffices at later TOFs.

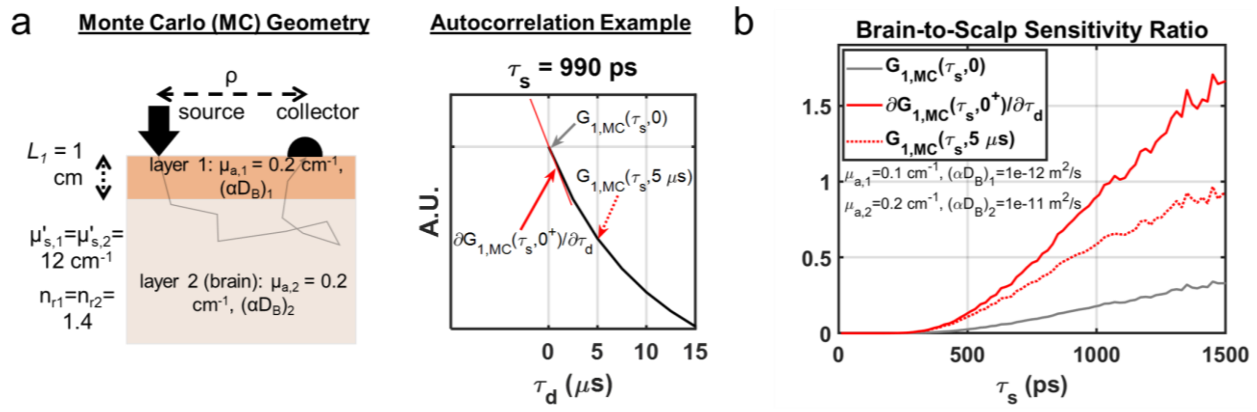

Supplementary Figure 13. Brain-to-scalp sensitivity. **a**, Simulation geometry, with a 1 cm thick extracerebral layer (representing mainly scalp and skull) and a semi-infinite layer representing the brain. All simulations were performed at null SD separation (ρ=0 cm), with the optical properties shown. An example simulated autocorrelation at τ_s=990 ps is shown, with 3 candidate parameters to achieve brain sensitivity. **b**, TOF-resolved brain-to-scalp sensitivity ratio for TOF-resolved intensity with absorption changes (solid gray), as is measured in time-domain NIRS, and TOF-resolved autocorrelation features with dynamics changes (red), as measured in iNIRS. Two different autocorrelation parameters were considered: the autocorrelation slope at zero time lag (best case, solid red), and the autocorrelation at half the lag time resolution (more realistic case, dotted red).

Supplementary Note 1: Bulk phase shifts distort the field autocorrelation but can be corrected

Besides the standard motion artifacts in NIRS, non-contact iNIRS is also susceptible to bulk phase shifts (bps), resulting from a change in the optical path to the sample. (Homodyne methods such as DWS/DCS are less susceptible to bulk phase shifts, since all sample paths are similarly affected.) In general, if $\mathbf{k_i}$ is the wave vector for the light incident on the sample from the source, $\mathbf{k_r}$ is the wave vector for the light remitted from the sample to the detector, and t_d is time delay, the cumulative phase is $\theta_{bps}(t_d)=\mathbf{k_r}\cdot\mathbf{r}(t_d)-\mathbf{k_i}\cdot\mathbf{r}(t_d)=\mathbf{q}\cdot\mathbf{r}(t_d)$, where $\mathbf{q}=\mathbf{k_r}-\mathbf{k_i}$ is the momentum transfer and $\mathbf{r}(t_d)$ is the relative displacement vector. In a double-pass back-reflectance geometry, the phase shift is $\theta_{bps}(t_d)=4\pi Z(t_d)/\lambda_0$, where λ_0 is the wavelength in free space and $Z(t_d)$ is the axial displacement of the sample towards the probe. Thus the measured mutual coherence function is given by $\Gamma'_{rs}(\tau_s,t_d)=\Gamma_{rs}(\tau_s,t_d)\exp[j\theta_{bps}(t_d)]$, where $\Gamma_{rs}(\tau_s,t_d)$ is the mutual coherence function (excluding the bulk phase shift) and $j=\sqrt{-1}$. Under the reasonable assumption that bulk phase shifts are independent of the intrinsic sample dynamics, the resulting iNIRS field autocorrelation (Eq. (1) in the main text) can be shown to be

$$G_1^{'iNIRS}(\tau_s,\tau_d)=G_1^{iNIRS}(\tau_s,\tau_d)G^{bps}(\tau_d), \quad (1)$$

where $G^{bps}(\tau_d)=<\exp[j\theta_{bps}(t_d+\tau_d)]\exp[-j\theta_{bps}(t_d)]>$ is the autocorrelation of the bulk phase shifts and brackets denote expectation or averaging over t_d. First, we note that since $G^{bps}(0)=1$, bulk phase shifts do not affect the TPSF, $I^{iNIRS}(\tau_s)=G_1^{iNIRS}(\tau_s,0)$. Second, if the time scale of bulk phase shifts (the decorrelation time of G^{bps}) is comparable to or less than the time scale of intrinsic field dynamics (the decorrelation time of G_1^{iNIRS}), then the autocorrelation is distorted. We empirically observe that G^{bps} exhibits a Gaussian decay (green in Figure 2.f, and Supplementary Figure 2.b). In our preliminary work, this initially led us to conclude (incorrectly) that intrinsic autocorrelations agreed with a random flow model at early TOFs[1]. Finally, autocorrelations at long TOFs can be estimated accurately even without correction of bulk phase shifts if the time scale of intrinsic field dynamics is sufficiently fast to dominate the autocorrelation decay in Supplementary Eq. (7) (i.e. long TOFs or large SD separation). To determine bulk phase shifts experimentally from iNIRS data, larger windows reduce noise in the estimation. However, time windows as short as two iNIRS measurements can be used, if signal-to-noise-ratio is sufficient and detection is performed at null SD. In this case, the phase shift can be estimated via a simple Kasai algorithm. Bulk

phase shifts are corrected by multiplying the measured mutual coherence function, $\Gamma'_{rs}(\tau_s,t_d)$, by exp[-j$\theta_{bps}(t_d)$], where $\theta_{bps}(t_d)$ is the bulk phase shift.

Supplementary Note 2: Predicting g_2 from g_1 and the modified Siegert relationship

To assess the effectiveness of motion correction, two methods of determining the intensity autocorrelation (g_2) were compared in Supplementary Figure 2.c. In the first method, which is sensitive to bulk phase shifts, the intensity autocorrelation was predicted from the iNIRS field autocorrelation and the modified Siegert relationship, as stated in Eq. (6) of Ref [2]. The fraction of static scattering, $\eta^{iNIRS}(\tau_s)$, and the dynamic autocorrelation component, $\gamma_1^{iNIRS}(\tau_s,\tau_d)$, as given in Eq. (5) of Ref. [2], were estimated from $g_1^{iNIRS}(\tau_s,\tau_d)$. In the second method, which is insensitive to bulk phase shifts, the intensity autocorrelation was determined directly from $|\Gamma_{rs}(\tau_s,\tau_d)|^2$, as described in Eq. (15) of Ref. [2], with correction for noise bias[2] for odd and even lags. The comparison in Supplementary Figure 2.c shows that motion invalidates the modified Siegert relationship, but our bulk phase correction method restored its validity.

Supplementary Note 3: Field autocorrelation simulations based on modified Bonner and Nossal theory

To show the plausibility of TOF-resolved autocorrelation characteristics measured by iNIRS, we start with a time-tested physical model put forth by Bonner and Nossal[3], which accounts for advection of red blood cells (RBCs), and adapt it to also include Brownian RBC motion. The fundamental unit in this model is a single dynamic scattering event from an RBC in a surrounding static tissue matrix that serves to randomize the incident direction and achieves an effective 4π illumination of the RBC (Supplementary Figure 7). A time-dependent phase shift occurs from the RBC scattering event while time-independent phase shifts (at least over time scales of interest) occur from the tissue matrix scattering events. Hence the time scale of decorrelation is determined by phase shifts from the RBC scattering event.

In practice, the detected electric field is the sum over many independent photon paths, each of which involves a different RBC scattering angle θ and displacement $\mathbf{\Delta r}$. The experimentally-estimated normalized autocorrelation is effectively an average over these paths and over time. If this average is representative of the ensemble, the autocorrelation estimated from measurements is equivalent to the ensemble-averaged autocorrelation. There are two ensemble averages implicit in this path summation: 1) an average over the

RBC displacement distribution and 2) an average over RBC scattering angle. If RBC motion and scattering angle are independent, ensemble averaging may be performed in sequence, in any order, using marginal displacement and scattering angle probability densities. Assuming a scattering phase function P(θ), the single scattering (ss) autocorrelation is given by:

$$g_1^{ss}(\tau_d) = \left\langle \left\langle \exp\left[jq(\theta) \bullet \Delta r(\tau_d) \right] \right\rangle_{\Delta r} \right\rangle_q = \int_0^{\pi} P(\theta) \left\langle \exp\left[jq(\theta) \bullet \Delta r(\tau_d) \right] \right\rangle_{\Delta r} 2\pi \sin(\theta) d\theta \, . \qquad (2)$$

Performing the inner ensemble average first, and assuming a three-dimensional Gaussian displacement distribution with variance $\left\langle \Delta r^2(\tau_d) \right\rangle = \left\langle \left| \Delta r(\tau_d) \right|^2 \right\rangle$, the autocorrelation is given by:

$$\left\langle \exp\left[jq(\theta) \bullet \Delta r(\tau_d) \right] \right\rangle_{\Delta r} = \exp\left[-\frac{q^2(\theta)}{6} \left\langle \Delta r^2(\tau_d) \right\rangle \right], \qquad (3)$$

where the magnitude of momentum transfer is given by

$$q(\theta) = \left| q(\theta) \right| = \frac{4\pi n_r \sin(\theta/2)}{\lambda_0} . \qquad (4)$$

For a Gaussian RBC velocity distribution with variance v^2, in addition to independent Brownian motion with effective Brownian coefficient D_B, it can be shown that

$$\left\langle \Delta r^2(\tau_d) \right\rangle = v^2 \tau_d^{\,2} + 6 D_B \tau_d \, . \qquad (5)$$

More generally, including the collision time scale, τ_C, to establish Brownian motion, we obtain:

$$\left\langle \Delta r^2(\tau_d) \right\rangle = v^2 \tau_d^{\,2} + 6 D_B \left\{ \tau_d - \tau_C \left[1 - \exp(-\tau_d / \tau_C) \right] \right\} . \qquad (6)$$

Interestingly, the collision time scale prior to establishment of Brownian motion results in τ_d^2 dependence for small $\tau_d << \tau_C$, while advection also results in τ_d^2 dependence for $\tau_d >> 6D_B/v^2$. Note that if there were only a single scattering angle θ (as is the case in dynamic light scattering), the form of the autocorrelation would agree with Eq. (5) in the main text. We expect DLS to hold only at the earliest TOFs and short SD separations. Otherwise, the randomization of light caused by the surrounding tissue matrix requires an average over RBC scattering angles as described in Supplementary Eq. (2). This angular average critically depends on the scattering phase function, P(θ), and may lead to an autocorrelation functional dependence on time lag that is distinctly different in form from Eq. (5) in the main text. In this work, the angular averaging is performed numerically, and the RBC is approximated with a Henyey-Greenstein phase function with anisotropy g_{RBC}.

To account for multiple scattering (ms) at later TOFs, we assume that dynamic scattering events constitute a Poisson process with time rate r. The mean number of dynamic scattering events is $n_{avg}=r\tau_s$. Following Bonner and Nossal, we assume that phase shifts from all dynamic scattering events are mutually uncorrelated. While this assumption holds for phase shifts due to diffusion, recent work has also suggested that the model might apply even if advection-induced phase shifts from dynamic scattering events are correlated[4]. Thus, the total multiple scattering autocorrelation, accounting for photon paths with different numbers of dynamic scattering events, is a weighted power series of single scattering autocorrelations:

$$g_1^{ms}(\tau_s,\tau_d)=\sum_{n=0}^{\infty}\frac{e^{-r\tau_s}(r\tau_s)^{-n}}{n!}\left[g_1^{ss}(\tau_d)\right]^n. \tag{7}$$

Finally, we also compare our physical model to a common diffusing wave spectroscopy (DWS) expression based on the cumulant approximation (i.e. the first cumulant expansion of the exponential). In this approximation, which is valid for large $r\tau_s$ and small τ_d, we neglect the variance in the number of dynamic scattering events between different photon paths, and we bring the ensemble average over scattering angle into the exponent:

$$g_1^{DWS}(\tau_s,\tau_d)\approx\left[g_1^{ss}(\tau_d)\right]^{r\tau_s}\approx\exp\left[-\frac{\langle q^2(\theta)\rangle}{6}\langle\Delta r^2(\tau_d)\rangle r\tau_s\right]=\exp\left[-\frac{k^2(1-g_{RBC})}{3}\langle\Delta r^2(\tau_d)\rangle r\tau_s\right]. \tag{8}$$

The rightmost expression is obtained from the relationship $\langle q^2(\theta)\rangle=2k^2(1-g_{RBC})$.This expression can be converted to the classical DWS expression (Eq. (6) in the main text) by substituting $\alpha\mu_s'L/(1-g_{RBC})$ for $r\tau_s$, where $\alpha=\mu_{s,RBC}(1-g_{RBC})/\mu_s'$, and $\mu_{s,RBC}$ is the scattering coefficient of RBCs in tissue, which may be approximated by the volume fraction of blood times the scattering coefficient of blood. For a 3% blood volume fraction and a blood scattering coefficient[5] of 70 mm^{-1} we estimate that $\mu_{s,RBC}$=2.1 mm^{-1} and r=0.45 ps^{-1}.

Several remarks are in order. First, in the cumulant approximation, the early decay behavior of the autocorrelation is determined only by the product of $\mu_{s,RBC}$, the RBC scattering coefficient, and $1-g_{RBC}$, the RBC scattering anisotropy, but is not influenced by either of these parameters individually, or the specific shape of the RBC scattering phase function. This represents a similarity relation for dynamic scattering that accompanies the cumulant approximation. For paths with more dynamic scattering events, the cumulant expansion (Supplementary Eq. (8)) becomes valid at larger time lags, and in the DWS limit, the

cumulant expansion is assumed to be universally valid over time lags of interest[6]. (Note that the common practice of performing Monte Carlo simulations with bulk tissue optical properties, then incorporating an effective diffusion coefficient of αD_B instead of D_B to assess dynamics[7], implicitly assumes the cumulant approximation.) Second, from Supplementary Eq. (5), Brownian motion dominates the displacement variance for time lags shorter than $6D_B/v^2$. Thus, importantly, the asymptotic DWS form of the autocorrelation for large TOFs and early time lags, which depends on the early lag time behavior of $g_1^{ss}\left(\tau_d\right)$, must be dominated by Brownian motion, even though RBC advection is present. The field associated with small angle dynamic scattering events decorrelates much slower than the field from large angle dynamic scattering events. Yet for typical RBC scattering phase functions with high anisotropy[8], small angle dynamic scattering events are highly probable. For a Henyey-Greenstein phase function with g_{RBC}=0.975, 90% of the scattering events occur at polar angles of θ≤13°. As shown in Supplementary Figure 8, these slowly decorrelating small angle paths lead to a "long tail" in the measured autocorrelation function, which persists even after many dynamic scattering events. In particular, paths that consist only of small angle dynamic scattering events dominate at long autocorrelation lags. The overall behavior, for both a pure Brownian motion model as well as a hybrid model, is well-described by a bi-exponential fit with a rapid and slow decay (Supplementary Figure 8.b-c). Notably, the slow component is attenuated and the cumulant approximation is improved if the scattering anisotropy is reduced. After just 10 dynamic scattering events, the cumulant approximation provides an excellent description of simulated autocorrelations from diffusing Intralipid (Supplementary Figure 9.a) but not from more anisotropically scattering dynamic red blood cells (Supplementary Figure 9.b). These simulations explain our experimental observation that a mono-exponential fit suffices for Intralipid (Figure 3), but not in real vascular beds (Figure 4). Interestingly, the cumulant approximation can be improved, even without changing anisotropy at all, by merely changing the shape of the dynamic scattering phase function to mitigate small angle scattering (Supplementary Figure 10). Based on this wealth of evidence, we associate the presence of the slow component with dynamical snake paths (Supplementary Figure 11.b), paths that consist only of small angle dynamic scattering events. While a hybrid model with early ballistic motion qualitatively predicted the observed autocorrelation behavior (Supplementary Figure 12), further work is required to ascertain the best model fit. One important question is the appropriate RBC scattering phase function. Even for scattering phase functions with the same

anisotropy (g_{RBC}) value, the decay behavior of the autocorrelation can be distinctly different (Supplementary Figure 10). For instance, the Henyey-Greenstein phase function (equivalent to the Gegenbauer kernel phase function with $\alpha_{Geg.}$=0.5) predicts a much slower autocorrelation tail than a Gegenbauer kernel phase function with $\alpha_{Geg.}$=1, even with the same g_{RBC}. Second, as RBCs orient and deform in shear flow, there may be a correlation between scattering angle and RBC displacement, contrary to our assumptions above. Third, the contribution of rotational dynamics, which is potentially significant given the high scattering anisotropy of RBCs, remains unknown. Finally, dynamical snake paths and layers with heterogeneous dynamics can both lead to non-exponential autocorrelations. Indeed, averaging to reduce noise at larger TOFs (Supplementary Figure 4) suggests that a bi-exponential decay is observed well past 500 ps in iNIRS of the human forehead. This finding cannot be plausibly attributed to dynamical snake paths, and likely represents the divergent dynamics of extracerebral and cerebral layers, as discussed in Supplementary Note 5.

Supplementary Note 4: Synthesizing DWS/DCS from iNIRS

To decipher classical TOF-integrated field autocorrelations from DWS/DCS (Figure 6), we must synthesize DWS/DCS-like autocorrelations from the bottom up, i.e. integrating iNIRS field autocorrelations over TOF:

$$G_1^{DCS}(\tau_d) = \int_{-\infty}^{\infty} G_1^{iNIRS}(\tau_s, \tau_d)\, d\tau_s \ , \qquad (9)$$

This is similar to integrating the iNIRS mutual coherence function over TOF, prior to autocorrelation, and (nearly) equivalent to evaluating the autocorrelation of the interference fringes at each optical frequency and averaging directly.

Supplementary Note 5: Field autocorrelation simulations based on cumulant approximation for correlation transport

To provide context for our TOF-resolved measurements in multilayered tissues with heterogeneous optical properties and dynamics, we performed Monte Carlo simulations to record both partial TOF (proportional to partial path length) and partial momentum transfer in a two-layer model of the human head. Unlike the Bonner and Nossal simulations described above, the Monte Carlo simulations did not account for either deviations from the cumulant approximation or the presence of ordered motion. Thus, this simulation is expected to be most accurate at early time lags, as well as late times-of-flight. We used the GPU-based

Monte Carlo eXtreme (MCX) code developed by Fang et al[9], setting both source and detector radii to 1 mm. For the simulation, we used dual NVIDIA GeForce RTX 2080 GPUs, storing 50 million detected photons at null source-detector separation (ρ=0 cm). The thickness of the extracerebral layer (layer 1) was set to 1 cm while the cerebral layer (layer 2) was semi-infinite. The reduced scattering coefficient and index of refraction were 12 cm^{-1} and 1.4, respectively, for both layers (Supplementary Figure 13.a). The simulation returns a list of all detected photons, and their individual partial path lengths (L_1 and L_2) and partial (dimensionless) momentum transfers (Y_1 and Y_2) in each tissue type. Absorption was included by weighting each photon according to partial path length, and the respective medium absorption coefficient ($\mu_{a,1}$=0.1 cm^{-1} for the extracerebral layer and $\mu_{a,2}$=0.2 cm^{-1} for the cerebral layer), by applying the Beer-Lambert law. Photons were binned into 20 ps TOF windows; autocorrelations, $G_{1,MC}(\tau_s,\tau_d)$, were calculated for each TOF window assuming the cumulant approximation and blood flow indices $(\alpha D_B)_1$ and $(\alpha D_B)_2$ for the two layers, based on the following expression:

$$G_{1,MC}\left(\tau_s,\tau_d\right) = I_{no\ abs.}\left(\tau_s\right)\times \int_{L_2}\int_{L_1}\int_{Y_2}\int_{Y_1} f\left(L_1,L_2,Y_1,Y_2|\tau_s\right)\exp\left(-\mu_{a,1}\ L_1\right)\exp\left[-2k^2\left(\alpha D_B\right)_1 Y_1\tau_d\right]\exp\left(-\mu_{a,2}\ L_2\right)\exp\left[-2k^2\left(\alpha D_B\right)_2 Y_2\tau_d\right]dY_1dY_2dL_1dL_2 \quad (10)$$

In practice, the integral is approximated by summing over all detected photons in the Monte Carlo simulation. Note that f is the probability density of partial path lengths and momentum transfers assuming zero absorption, given TOF, τ_s, and $I_{no\ abs.}(\tau_s)$ is the medium photon TOF distribution (DTOF) assuming zero absorption. First, to simulate brain-to-scalp sensitivity, we considered three different parameters which depend on TOF, or τ_s (Supplementary Figure 13.a). For a standardized comparison we considered the autocorrelation at zero time lag (i.e. the intensity), $I(\tau_s)=G_{1,MC}(\tau_s,0)$, which is measured in conventional time-domain NIRS. For this parameter, the brain-to-scalp sensitivity was taken as $[\partial I(\tau_s)/\partial\mu_{a,2}\times\mu_{a,2}]$ / $[\partial I(\tau_s)/\partial\mu_{a,1}\times\mu_{a,1}]$. For field fluctuations, we considered two parameters denoted $p(\tau_s)$: a "best case" scenario $p(\tau_s)=\partial G_{1,MC}(\tau_s,\tau_d=0^+)/\partial\tau_d$, which is the slope of the autocorrelation at zero time lag, and a more realistic scenario $p(\tau_s)=G_{1,MC}(\tau_s,\tau_d=5\ \mu s)$, which is the change in the autocorrelation at half of our experimental lag time resolution of 10 μs. For fluctuation measurements, the brain-to-scalp sensitivity was taken as $[\partial p(\tau_s)/\partial(\alpha D_B)_2\times(\alpha D_B)_2]$/ $[\partial p(\tau_s)/\partial(\alpha D_B)_1\times(\alpha D_B)_1]$. To simulate TOF-resolved autocorrelations for determining the sensitivity, we used standard values from literature of $(\alpha D_B)_1=1\times10^{-12}$ m^2s^{-1} for the superficial layer and

$(\alpha D_B)_2=1\times10^{-11}$ m^2s^{-1} for brain dynamics[10]. The simulations suggest a brain-to-scalp sensitivity ratio of ~0.2 for classical TOF-resolved intensity measurements at 1 ns, while the iNIRS brain-to-scalp sensitivity ratio, based on field fluctuations at 1 ns, is at least two times higher (Supplementary Figure 13.b).

Second, in Supplementary Figure 5, we modified the simulations by choosing $(\alpha D_B)_1$ and $(\alpha D_B)_2$ to yield agreement with experimental brain data at baseline (Figure 5.f). The resultant blood flow indices of $(\alpha D_B)_1=0.5\times10^{-12}$ m^2s^{-1} for the superficial layer and $(\alpha D_B)_2=1.5\times10^{-11}$ m^2s^{-1} for brain are in rough agreement with standard values[10], taking into account that null SD measurements are susceptible to heterogeneities in scalp blood flow at early TOFs. The results of the simulation and parameters from a 5-parameter fit (Eq. (8) in the main text) are shown in Supplementary Figure 5.a-b. Third, without modifying the assumed blood flow indices, we investigated the hypothesis that sampling of tissues with differing flows could lead to a biexponential autocorrelation decay. TOF-resolved autocorrelations were fitted by 3-parameter (Eq. (7) in the main text) and 5-parameter (Eq. (8) in the main text) models, and the adjusted R^2 values are compared in Supplementary Figure 5.c. Interestingly, the 3-parameter model provided good fits until about 500 ps, when brain sensitivity begins to increase and systematic deviations became apparent, necessitating the 5-parameter fit. On the other hand, when the two layers were assumed to have the same blood flow, the 5-parameter fit was not required at any TOFs (data not shown). Thus, we conclude that sampling of tissues with heterogeneous dynamics can also lead to a bi-exponential autocorrelation decay. This provides further support for the conclusions summarized in Table 1.

Supplementary Note 6: Proof of iNIRS convolution expression [Eq. (2) in the main text]

The iNIRS mutual coherence function, Γ_{rs}, can be expressed as the intrinsic complex field distribution of the sample, Γ_0, convolved in TOF with the point spread function (PSF):

$$\Gamma_{rs}\left(\tau_s,t_d\right)=\Gamma_0\left(\tau_s,t_d\right)*\mathrm{PSF}\left(\tau_s\right). \tag{11}$$

We assume that the complex signal includes a fluctuating, zero mean contribution, $\Gamma_{0,f}$, caused by paths with momentum transfer at dynamic scattering events, as well as a constant contribution, $\Gamma_{0,c}$:

$$\Gamma_0\left(\tau_s,t_d\right)=\Gamma_{0,f}\left(\tau_s,t_d\right)+\Gamma_{0,c}\left(\tau_s\right). \tag{12}$$

We assume that the fluctuating field comprises multiple uncorrelated photon paths[3]. Thus the fluctuating field is uncorrelated at different TOFs, i.e.

$$\left\langle \Gamma^{*}_{0,f}\left(\tau_s, t_d\right)\Gamma_{0,f}\left(\tau_s^{'}, t_d+\tau_d\right)\right\rangle_{t_d} = G_{1,f}\left(\tau_s,\tau_d\right)\delta\left(\tau_s-\tau_s^{'}\right). \tag{13}$$

Including the constant component, we obtain:

$$\left\langle \Gamma^{*}_{0}\left(\tau_s, t_d\right)\Gamma_{0}\left(\tau_s^{'}, t_d+\tau_d\right)\right\rangle_{t_d} = \Gamma^{*}_{0,c}\left(\tau_s\right)\Gamma_{0,c}\left(\tau_s^{'}\right)+G_{1,f}\left(\tau_s,\tau_d\right)\delta\left(\tau_s-\tau_s^{'}\right). \tag{14}$$

Thus, by plugging Supplementary Eq. (11) into the expression for the iNIRS autocorrelation (Eq. (2) in the main text) and applying Supplementary Eq. (14), we obtain

$$G_1^{iNIRS}\left(\tau_s,\tau_d\right) = \left|\Gamma_{0,c}\left(\tau_s\right) * PSF\left(\tau_s\right)\right|^2 + G_{1,f}\left(\tau_s,\tau_d\right) * IRF\left(\tau_s\right), \tag{15}$$

where $IRF(\tau_s)=|PSF(\tau_s)|^2$. The non-ergodic component, given by the first term in Supplementary Eq. (15), presents a challenge. In prior work, we have considered two cases where Supplementary Eq. (15) can be simplified: 1) the constant component is confined to a range of TOFs much narrower than the PSF (as in ballistic transmission)[2], and 2) temporal averaging, assumed to be equivalent to ensemble averaging, is achieved by lengthening the measurement time to satisfy ergodicity[11]. In case 2, which applies here, we may simplify Supplementary Eq. (15) as:

$$G_1^{iNIRS}\left(\tau_s,\tau_d\right) = I_c\left(\tau_s\right) * IRF\left(\tau_s\right) + G_{1,f}\left(\tau_s,\tau_d\right) * IRF\left(\tau_s\right). \tag{16}$$

(Note that even in case 1, Supplementary Eq. (16) may also be used, if we define $I_c(\tau_s)=|\int\Gamma_{0,c}(\tau_s)d\tau_s|^2\delta(\tau_s-\tau_{s,b})$ where $\tau_{s,b}$ is the ballistic time). In this work, $I_c=\langle|\Gamma_{0,c}|^2\rangle$ refers to a very slowly decorrelating, but still ergodic, component of the field autocorrelation which most likely corresponds to paths with scattering events from extravascular tissue.

Supplementary References

1. Kholiqov, O., Zhou, W. & Srinivasan, V. J. Interferometric near-infrared spectroscopy (iNIRS) at short source-detector separations (Conference Presentation). in *Neural Imaging and Sensing 2018* vol. 10481 104810X (International Society for Optics and Photonics, 2018).
2. Borycki, D., Kholiqov, O. & Srinivasan, V. J. Interferometric near-infrared spectroscopy directly quantifies optical field dynamics in turbid media. *Optica* **3**, 1471–1476 (2016).
3. Bonner, R. & Nossal, R. Model for laser Doppler measurements of blood flow in tissue. *Appl. Opt.* **20**, 2097–2107 (1981).
4. Sakadžic, S., Boas, D. A. & Carp, S. Theoretical model of blood flow measurement by diffuse correlation spectroscopy. *J. Biomed. Opt.* **22**, 27006 (2017).

5. Bosschaart, N., Edelman, G. J., Aalders, M. C. G., van Leeuwen, T. G. & Faber, D. J. A literature review and novel theoretical approach on the optical properties of whole blood. *Lasers Med. Sci.* **29**, 453–479 (2014).
6. Pine, D. J., Weitz, D. A., Zhu, J. X. & Herbolzheimer, E. Diffusing-wave spectroscopy: dynamic light scattering in the multiple scattering limit. *J. Phys.* **51**, 2101–2127 (1990).
7. Sathialingam, E. *et al.* Small separation diffuse correlation spectroscopy for measurement of cerebral blood flow in rodents. *Biomed. Opt. Express* **9**, 5719–5734 (2018).
8. Hammer, M., Schweitzer, D., Michel, B., Thamm, E. & Kolb, A. Single scattering by red blood cells. *Appl. Opt.* **37**, 7410–7418 (1998).
9. Fang, Q. & Boas, D. A. Monte Carlo Simulation of Photon Migration in 3D Turbid Media Accelerated by Graphics Processing Units. *Opt. Express* **17**, 20178–20190 (2009).
10. Baker, W. B. *et al.* Pressure modulation algorithm to separate cerebral hemodynamic signals from extracerebral artifacts. *Neurophotonics* **2**, 035004 (2015).
11. Borycki, D., Kholiqov, O. & Srinivasan, V. J. Reflectance-mode interferometric near-infrared spectroscopy quantifies brain absorption, scattering, and blood flow index in vivo. *Opt. Lett.* **42**, 591–594 (2017).
