# Supplementary material for: Time-of-flight resolved light field fluctuations reveal deep human tissue physiology
Source: Nat Commun. 2020 Jan 20;11:391. doi: 10.1038/s41467-019-14228-5 (PMC6971031; doi:10.1038/s41467-019-14228-5)
Supplement: Supplementary file 2 — Reporting Summary [file 41467_2019_14228_MOESM2_ESM.pdf]

nature research

Corresponding author(s): Oybek Kholiqov

Last updated by author(s): Nov 9, 2019

Reporting Summary

Nature Research wishes to improve the reproducibility of the work that we publish. This form provides structure for consistency and transparency in reporting. For further information on Nature Research policies, see Authors & Referees and the Editorial Policy Checklist .

Statistics

For all statistical analyses, confirm that the following items are present in the figure legend, table legend, main text, or Methods section.

n/a	Confirmed	
☐	☒	The exact sample size (n) for each experimental group/condition, given as a discrete number and unit of measurement
☐	☒	A statement on whether measurements were taken from distinct samples or whether the same sample was measured repeatedly
☐	☒	The statistical test(s) used AND whether they are one- or two-sided<br>*Only common tests should be described solely by name; describe more complex techniques in the Methods section.*
☐	☒	A description of all covariates tested
☐	☒	A description of any assumptions or corrections, such as tests of normality and adjustment for multiple comparisons
☐	☒	A full description of the statistical parameters including central tendency (e.g. means) or other basic estimates (e.g. regression coefficient) AND variation (e.g. standard deviation) or associated estimates of uncertainty (e.g. confidence intervals)
☐	☒	For null hypothesis testing, the test statistic (e.g. F, t, r) with confidence intervals, effect sizes, degrees of freedom and P value noted<br>*Give P values as exact values whenever suitable.*
☒	☐	For Bayesian analysis, information on the choice of priors and Markov chain Monte Carlo settings
☒	☐	For hierarchical and complex designs, identification of the appropriate level for tests and full reporting of outcomes
☒	☐	Estimates of effect sizes (e.g. Cohen's d, Pearson's r), indicating how they were calculated

Our web collection on statistics for biologists contains articles on many of the points above.

Software and code

Policy information about availability of computer code

Data collection	A LabVIEW software development kit (SDK) was purchased from GaGe (manufacturer of digitizer) and used to create a custom acquisition software for all measurements.
Data analysis	All data analysis was performed in MATLAB.

For manuscripts utilizing custom algorithms or software that are central to the research but not yet described in published literature, software must be made available to editors/reviewers. We strongly encourage code deposition in a community repository (e.g. GitHub). See the Nature Research guidelines for submitting code & software for further information.

Data

Policy information about availability of data

All manuscripts must include a data availability statement. This statement should provide the following information, where applicable:

- Accession codes, unique identifiers, or web links for publicly available datasets
- A list of figures that have associated raw data
- A description of any restrictions on data availability

All data supporting the findings reported in this study are available in the article itself and supplementary information document. Further inquiries regarding the reproduction of published findings are available from the corresponding author upon reasonable request.

Field-specific reporting

Please select the one below that is the best fit for your research. If you are not sure, read the appropriate sections before making your selection.

☒ Life sciences ☐ Behavioural & social sciences ☐ Ecological, evolutionary & environmental sciences

October 2018

Life sciences study design

All studies must disclose on these points even when the disclosure is negative.

Sample size	Our mouse IACUC protocol states: numbers were estimated based on our pilot studies where we experienced a standard deviation of approximately 14% of the mean. According to this, in order to detect a 10% change in measured parameters (i.e. vessel dilation or contraction, neuronal activation above baseline), we need group sizes of 20 animals including 10% failure rate. Power analysis as follows; alpha=0.05, two-tailed, power=0.8.<br><br>For human studies, our IRB protocol states: a two-sided equality test with a statistical power of 0.8 and type I error rate of 0.05, assuming a standard deviation equal to 25% of the mean requires a sample size of 13 subjects. To allow for a 10% experimental failure rate (physiology out of range, low signal, etc.), we increase the total number of subjects for the pilot study to 15 subjects.<br><br>Ultimately the data described in this paper come from 3 mice and 3 human subjects, which are smaller than the target enrollments in our ongoing protocols. The purpose of the paper is to establish the demonstration, fundamental physics, and interpretation of our novel iNIRS technique, and subsequent works will establish variability across the population of our novel physiological measurements.
Data exclusions	No data were excluded from the analyses.
Replication	All attempts at replication were successful.
Randomization	Randomization was not relevant to this study.
Blinding	Blinding was not relevant to this study.

Reporting for specific materials, systems and methods

We require information from authors about some types of materials, experimental systems and methods used in many studies. Here, indicate whether each material, system or method listed is relevant to your study. If you are not sure if a list item applies to your research, read the appropriate section before selecting a response.

Materials & experimental systems

n/a	Involved in the study	
☒	☐	Antibodies
☒	☐	Eukaryotic cell lines
☒	☐	Palaeontology
☐	☒	Animals and other organisms
☐	☒	Human research participants
☒	☐	Clinical data

Methods

n/a	Involved in the study	
☒	☐	ChIP-seq
☒	☐	Flow cytometry
☒	☐	MRI-based neuroimaging

Animals and other organisms

Policy information about studies involving animals; ARRIVE guidelines recommended for reporting animal research

Laboratory animals	Nude mice (male, SKH-1E Charles River).
Wild animals	N/A
Field-collected samples	N/A
Ethics oversight	All experimental procedures and protocols were reviewed and approved by the UC Davis Institutional Animal Care and Use Committee.

Note that full information on the approval of the study protocol must also be provided in the manuscript.

Human research participants

Policy information about studies involving human research participants

Population characteristics	All male, age 28-30, no history of heart, lung, or blood related diseases.
Recruitment	The human subjects were recruited via a recruitment flyer approved by the UC Davis Institutional Review Board.

Ethics oversight	All protocols were approved by the UC Davis Institutional Review Board.

Note that full information on the approval of the study protocol must also be provided in the manuscript.
